# Therapeutic Potential of Ellagic Acid in Liver Diseases

**DOI:** 10.3390/molecules30122596

**Published:** 2025-06-15

**Authors:** Karolina Wojtunik-Kulesza, Przemysław Niziński, Anna Krajewska, Tomasz Oniszczuk, Maciej Combrzyński, Anna Oniszczuk

**Affiliations:** 1 Department of Inorganic Chemistry, Medical University of Lublin, Chodźki 4a, 20-093 Lublin, Poland; karolina.wojtunik-kulesza@umlub.pl; 2 Department of Pharmacology, Medical University of Lublin, Radziwiłłowska 11, 20-080 Lublin, Poland; przemyslaw.nizinski@umlub.pl; 3 Chair of Comprehensive Dentistry, Medical University of Lublin, Chodżki 6, 20-093 Lublin, Poland; anna.krajewska1@umlub.pl; 4 Department of Thermal Technology and Food Process Engineering, University of Life Sciences in Lublin, Głęboka 31, 20-612 Lublin, Poland; tomasz.oniszczuk@up.lublin.pl (T.O.); maciej.combrzynski@up.lublin.pl (M.C.)

**Keywords:** polyphenols, antioxidants, ellagic acid, urolithins, MASLD

## Abstract

Ellagic acid (EA) is a natural polyphenol found in various fruits, nuts, and mushrooms. It exhibits a variety of biological activities, including potent antioxidant, anti-inflammatory, anti-obesity, and neuroprotective properties. EA exerts hepatoprotective effects through multiple mechanisms, including (1) scavenging reactive oxygen species (ROS) and enhancing endogenous antioxidant defenses (e.g., by activating Nrf2/ARE), (2) modulating inflammatory signaling pathways (e.g., inhibiting NF-κB, TNF-α, and IL-6), and (3) regulating apoptosis (e.g., downregulating the Bax/Bcl-2 ratio) and fibrosis (e.g., inhibiting TGF-β/Smad signaling). Despite its promising preclinical efficacy, the clinical applicability of EA is currently limited by its poor bioavailability. This could potentially be overcome by advanced delivery systems or by directly administering its active microbial metabolites, known as urolithins. EA and its derivatives also modulate the gut microbiota, promoting the growth of beneficial species and reducing gut permeability and hepatic inflammation. Preliminary clinical trials and other emerging evidence suggest that EA may reduce liver inflammation, oxidative stress, and metabolic dysregulation. However, more extensive human studies are needed to confirm its efficacy and safety in managing liver disease. This review highlights the therapeutic potential of EA in the treatment of liver diseases, particularly metabolic-dysfunction-associated steatotic liver disease (MASLD).

## 1. Introduction

Throughout our lives, from day one, our bodies are exposed to attacks from harmful pro-radicals that lead to cell damage. In addition to endogenous defenses, externally supplied additional means of protection are extremely important. The most prominent source of active compounds are plants, fruits, and vegetables, which are both inexhaustible and a safe source of natural defenders for our organism. In the canon of the most important natural active compounds is ellagic acid (EA), one of the most active secondary plant metabolites that demonstrates a wide spectrum of biological activities [1].

**Figure 1:**
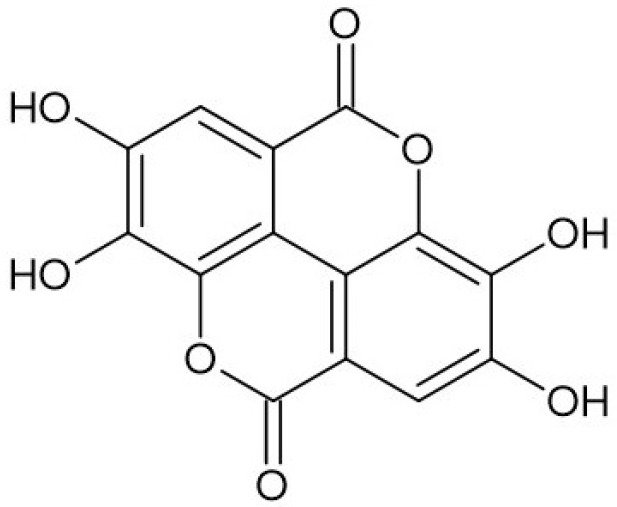
Structure of ellagic acid.

Ellagic acid (2,3,7,8-tetrahydroxy-chromeno[5,4,3-cde]chromene-5,10-dione), belonging to a chromene-dione derivative, consists of two characteristic regions: a hydrophilic region made up of four hydroxyl groups and two lactone rings, as well as a lipophilic region made up of two hydrocarbon rings [2] (Figure 1). The compound can be found in free form as well as part of ellagitannins, which are more complex structures. On the whole, ellagic acid can be found as odorless cream-colored needles or as a yellow powder, demonstrating strong reducing activity. EA can be found in numerous fruits (e.g., strawberry, goji berry, raspberry), nuts (e.g., chestnut, walnut), and mushrooms (e.g., *Fistulina hepatica*), whereas vegetables are a less prominent source of the compound [3,4]. Many ellagic acid sources have been studied in terms of their pro-health properties, confirming their antibacterial, hepatoprotective, antiviral, anti-inflammatory, neuroprotective, and many more functions, which are strictly associated with their high content of ellagic acid and its wide spectrum of biological activities [5,6].

The specific structure of ellagic acid, containing four hydroxyl and two lactone groups, is responsible for its high free radical scavenging and antioxidant activities [7]. The compound is as important as other significant antioxidants (e.g., ascorbic acid, vitamin E) [8]. The high antioxidant activity of the acid is based both on direct free radical scavenging, the inhibition of lipid peroxidation, as well as the enhancement of antioxidant enzyme activities such as superoxide dismutase, catalase, and glutathione peroxidase [9]. The literature also reports several other possible mechanisms of action of the acid, leading to a reduction in lipidemic profile and lipid metabolism, neuroprotective effects, as well as an impact on metabolic syndrome and diabetes [10]. Among the most often presented possible mechanisms are it being able to decrease the activity of nuclear factor-κB (NF-κB), increase nuclear factor erythroid 2-related factor 2 expression, and inhibit cytochrome P-450 and cyclooxygenase-2 (COX-2) [11].

A significant number of studies on EA characterization are based on its promising hepatoprotective activity. This feature is closely linked with its antioxidant activity, ability to scavenge free radicals, and ability to inhibit hepatic stellate cell activation [12]. Additionally, Kang et al. [13] underlined that ellagic acid is able to improve metabolic health by regulating lipid metabolism and attenuating obesity-mediated complications like non-alcoholic fatty liver disease. There are also studies based on impact of the EA on metabolic-dysfunction-associated steatotic liver disease (MASLD), evaluated using in vivo studies [14]. As it turns out, Zhang et al.’s research provided information about the positive impact of ellagic acid on alcohol-induced liver disease by reducing oxidative stress, along with an improvement in gut microbiota and anti-inflammatory activity [15]. It is important that, in many cases, the positive influence of the compound on hepatoprotection is strictly associated with its strong antioxidant, free radical scavenging, and anti-inflammatory activities. 

**Figure 2:**
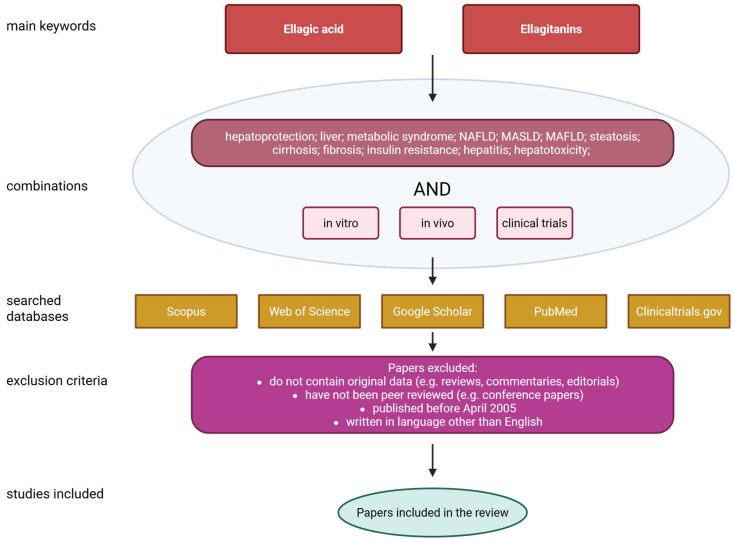
Flowchart of data collection. Created with BioRender^®^.

This review focuses on the detailed characterization of ellagic acid, its sources, biological functions, bioavailability and safety. A significant part of the paper is focused on the effect of EA on liver condition as well as MASLD. The paper also highlights the disadvantages associated with the poor solubility of ellagic acid in water, its short plasma half-life, and poor bioavailability after oral administration. Furthermore, it discusses several possible solutions that may help address these issues. The literature search was conducted using the PubMed, Scopus, Web of Science, Google Scholar and ClinicalTrials.gov databases. The following queries were used: ‘Ellagic acid’ or ‘Ellagitanins’ and ‘hepatoprotection’; ‘liver’; ‘metabolic syndrome’; ‘NAFLD’; ‘MASLD’; ‘MAFLD’; ‘steatosis’; ‘cirrhosis’; ‘fibrosis’; ‘insulin resistance’; ‘hepatitis’; ‘hepatotoxicity’. All of the above combinations were also supplied with the terms ‘in vitro’, ‘in vivo’ or ‘clinical trials’. Only papers published in the last twenty years (i.e., from April 2005 to April 2025) were included. To qualify for inclusion in the review, papers had to meet the following criteria: contain original data; have been independently reviewed; be written in English; and have been published after April 2005. Figure 2 shows a flowchart illustrating the data collection process for the review.

## 2. Sources and Biological Functions of Ellagic Acid

### 2.1. Sources of Ellagic Acid

As mentioned in the previous section, EA can be found in various fruits, nuts and mushrooms. Many analyses using common analytical methods such as chromatography allowed researchers to provide a quantitative determination of the acid in many plants [16]. Ellagitannins, which are found naturally in various plants and vegetables as hexahydroxydiphenoylglucose esters, undergo hydrolysis upon ingestion, releasing ellagic acid. This compound is only minimally absorbed in the stomach and small intestine [17]. The primary sources of EA, along with the most important data, are displayed in Table 1.

### 2.2. Biological Functions of Ellagic Acid

**Figure 3:**
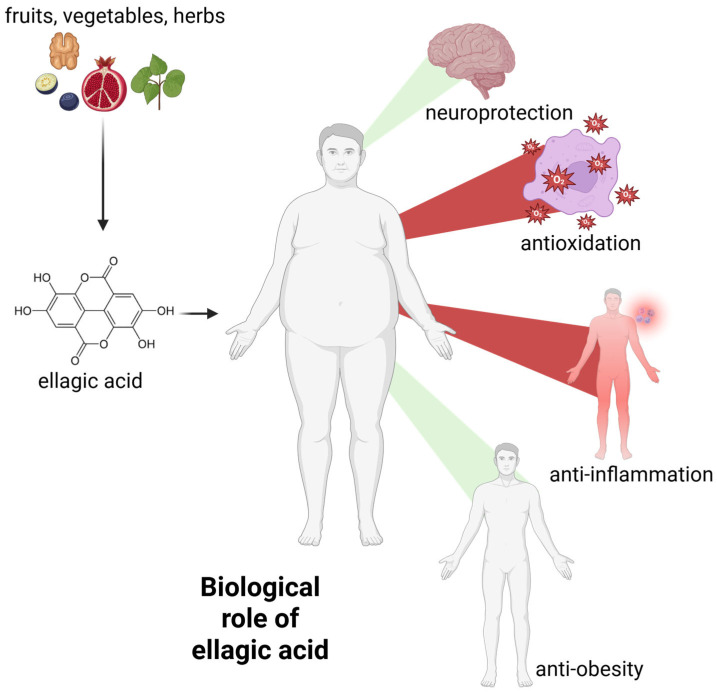
The general biological activity of ellagic acid. Green beams indicate the promotion of neuroprotection and weight loss, while red beams indicate the decrease in systemic inflammation and free radical levels. For further information, please see text below. Created with BioRender^®^.

Ellagic acid is a polyphenol with a wide range of biological activities. Free radical scavenging and antioxidant activity are among the most frequently and thoroughly studied mechanisms. These activities form the basis of others, such as anti-inflammatory processes [27,28]. Below we present the most important activities of EA. A schematic representation of the biological activity of EA is also presented in Figure 3.

#### 2.2.1. Antioxidant and Free Radical Scavenging Activity

The specific structure of EA, which contains four hydroxyl groups, is responsible for high antioxidant activity. This feature has been observed in both in vitro and in vivo studies. Some scientists have suggested that EA is a more effective antioxidant than vitamin E, a key compound used to combat oxidative stress and lipid peroxidation [29]. There are a number of possible mechanisms of action for EA, which is supported by concrete studies. Scientists indicate that the mechanism is strictly linked with a few factors such as polarity of reaction media, type of free radicals as well as deprotonated portion of EA [30]. Based on the studies presented by Mazzone et al. [31], the HAT (hydrogen atom transfer) mechanism can be observed in the case of a non-polar reaction environment, whereas the SPLET (sequential proton loss electron transfer) mechanism can be observed in a polar reaction environment. Tiwari and Mishra [6] made a significant contribution to the understanding of the antioxidant mechanism of ellagic acid (EA) by modeling its free radical scavenging activity against hydroxyl (^•^OH), methoxy (^•^OCH_3_), and nitrogen dioxide (^•^NO_2_) radicals.

The high free radical scavenging ability of EA was proven by Kumar et al. [32], who evaluated the activity using the common 1,1-diphenyl-2-picrylhydrazyl (DPPH) method, as well as cellular system. In both cases, high EA activity was recorded. It was also observed that EA can scavenge free radicals at a concentration of 1 µM over extended periods of time as well as inhibit the production of ROS in L-6 myoblasts and NADPH-dependent catalyzed microsomal lipid peroxidation. Density functional theory (DFT) used by Galano et al. [4] revealed that EA, especially its anion form, is able to protect against toxic effects of oxidative stress. It is also significant that the activity of EA is not reduced after its metabolism, making EA a particularly effective antioxidant. It is worth mentioning the multi-directional character of the compound. It is known that EA after deprotonation is able to chelate copper, leading to a reduction in free radical synthesis [4]. The high capacity of EA to reduce and chelate metal ions has been well established through extensive research. For instance, studies based on ions of Ni, Cu, Fe, Co, Mg, Ca and Mn have confirmed the ability of EA to form complexes with the ions along with facilitating their excretion from biological systems [33]. The high ability to form complexes with iron ions means that EA is an effective protector against free radical generation. It is associated with the inhibition of the Haber-Weiss/Fenton reaction, which involves iron ions, leading to the formation of harmful free radicals [34].

In addition to in vitro and theoretical studies based on the antioxidant activity of EA, its biological function was confirmed in in vivo studies numerous times. EA has been shown to modulate oxidative stress in rats and protect hepatocytes from oxidative stress in mice [35]. Pre-treatment with ellagic acid significantly increased the viability of HaCaT cells and reduced the production of reactive oxygen species (ROS) and malondialdehyde (MDA) induced by UVA exposure. Additionally, ellagic acid effectively protected against UVA-triggered DNA damage, as demonstrated by the comet assay. Treatment also notably decreased UVA-induced apoptosis in HaCaT cells by mitigating DNA fragmentation, mitochondrial dysfunction, endoplasmic reticulum (ER) stress, caspase-3 activation, as well as normalizing Bcl-2/Bax expression. Importantly, the antioxidant activity of ellagic acid was positively associated with elevated levels of heme oxygenase-1 (HO-1) and superoxide dismutase (SOD) expression [36].

#### 2.2.2. Anti-Inflammatory Activity

It is well known that inflammation, which can be caused by tissue injury or infection, is a key factor leading to the development of various diseases, including cancer. While most drugs are based on steroids and non-steroidal drugs (NSAIDs), many natural compounds have been found to have high ability to reduce inflammation [37].

The anti-inflammatory activity of EA has been confirmed by various experimental models. Among the possible mechanisms of action, strong antioxidant activity is presented as the most important. Scientists indicate various ways to counteract the inflammation process [38]. These include EA’s ability to reduce vascular permeability and neutrophil recruitment [39], decrease pro-inflammatory cytokine and prostaglandin E2 levels [40] as well as block signaling pathways such as NF-κB and STAT3 [41]. The activity of EA based on modulation of NF-κB has been presented in a few other papers, which confirms this line of reasoning [42]. Equally important are studies investigating EA modulation of cyclooxygenase-2 (COX-2) production, which revealed that EA has higher binding affinity that diclofenac, which is associated with the formation of four hydrogen bonds, while diclofenac is able to make only two [11]. Some of the anti-inflammatory mechanisms are still unknown. One example is the ability of EA to inhibit the tautomerase activity of MIF. One theory suggests that EA may exert its inhibitory effect by interfering with tautomerase activity or by binding to the tautomerase-active site of the cytokine [43]. Lin et al. [44] present a wide spectrum of possible EA anti-inflammatory mechanisms of action, focusing on in vitro and in vivo studies of the protective effect of EA on osteoarthritis. In an in vitro experiment, EA was found to suppress IL-1β-induced expression of inducible nitric oxide synthase (iNOS), COX-2, nitric oxide (NO), tumor necrosis factor-alpha (TNF-α), prostaglandin E2 (PGE2), and interleukin-6 (IL-6). Additionally, EA reduced the IL-1β-induced expression of matrix metalloproteinase-13 (MMP-13) and a disintegrin and metalloproteinase with thrombospondin motifs 5 (ADAMTS-5), while enhancing the expression of type II collagen and aggrecan.

The high ability of EA to counteract inflammation is a significant biological function which can be used in new drug design. Taking into account that the process accompanies disease states, it is important to use substances with a calming effect, both in prevention and in the treatment of advanced conditions [44].

#### 2.2.3. Neuroprotective Activity

Neuroprotection of natural compounds is one of the most intensively studied biological activities. Many of these features can be linked to other applications of the compounds, e.g., anti-inflammatory, antioxidant or metal ion chelation. It results from the fact that many diseases, including neurodegenerative, are strictly associated with micro-inflammation, oxidative stress and disturbances in the level of metal ions [45,46]. This fact means that one compound can be active in many directions and be used to prevent several diseases. The neuroprotective activity of EA is associated with its strong antioxidant and anti-inflammatory activity as well as ability for metal ion reduction and chelation, as mentioned previously. Considering the anti-neurodegenerative activity of EA, it is pivotal to characterize its enzyme inhibition ability, as well as selected factors that influence specific neurodegenerative disorders such as Alzheimer’s (AD) and Parkinson’s (PD) diseases [47].

A significant pathological process in AD is hyperphosphorylation, which is associated with activation and oxidation of NF-κB, as well as with lipid peroxidation, glycation end products and DNA damage [48]. The significant role of EA in the prevention and treatment of neurodegeneration is associated with the ability of the compound to regulate the production of inflammatory cytokines IL-1β and TNF-1α, leading to the protection of neurons and improving pathological changes in the brain [49]. Microglia are the primary immune cells of the central nervous system (CNS). Upon activation, they release various pro-inflammatory cytokines, anaphylatoxins, reactive oxygen species, and neurotoxic compounds, all of which can contribute to neuronal damage. Recent research indicates that activated microglia are also capable of producing proteolytic enzymes, which can degrade cellular components and impair neuronal function [50]. Studies have confirmed the ability of EA to reduce the number of microglia and inhibit pro-inflammatory cytokines [51]. The influence of EA on cholinergic dysfunctions was also determined using a rat model alongside scopolamine impairment [52]. Analysis towards glutathione (GSH) content, activity of SOD and catalase (CAT) along with the Morris water maze test explicitly revealed neuroprotective activity of EA. Despite the fact that the compound was not as active as donepezil, scientists have indicated its activity against cholinergic and its ability to alleviate oxidative stress. EA also reveals the potential to inhibit β-site amyloid precursor protein cleaving enzyme 1 (BACE1). Significant interactions with crucial amino acid residues creating BACE1 active site were observed using in silico analysis [53].

The valuable activity of EA was also noticed against Parkinson’s disease, a disorder associated with dopamine neurons [54]. Significant studies were performed using a 1-methyl-4-phenyl-1,2,3,6-tetrahydropyridine (MPTP) mouse model. EA administration caused a decrease in the activity of superoxide dismutase and catalase, leading to oxidative stress as well as an increase in a lipid peroxidation product—malondialdehyde. Pre-treatment with ellagic acid was found to protect dopaminergic neurons, preserve dopamine transporters, restore antioxidant enzymes, prevent glutathione loss, reduce lipid peroxidation and lower inflammatory markers, including COX-2 and iNOS [54].

#### 2.2.4. Anti-Obesity Activity

The scourge of obesity that has taken hold in our society is developing at an alarming rate. Unfortunately, it is affecting younger and younger people, adolescents and even children. Being overweight and obese is not just about how you look or feel; above all, it has a negative impact on the whole body and increases the risk of many diseases [55]. Therefore, it is extremely important to combat this phenomenon. Natural compounds that support weight loss processes are becoming increasingly popular. Recently, there has been a great deal of interest in EA, the properties of which for improving metabolism have been studied under various conditions, including in living organisms [56].

The first stage of studies, namely in vitro, is often based on adipocyte models and studies towards regulation of lipid metabolism. Studies presented by Mejia-Meza et al. [57] revealed that EA-containing raspberry altered adipocyte differentiation as well as reduced lipid accumulation in 3T3-L1 cells. The activity was confirmed several times using the same cells [58]. A significant number of studies are based on the impact of EA on fat cell formation as a crucial element in the mechanism of obesity, but more and more attention is paid to terminal stages of differentiation or lipogenesis. Analysis based on a radiolabeled precursor showed that EA has an important impact on reducing de novo lipogenesis and TG esterification [13]. At this point it is worth mentioning pomegranate, an important weight loss supplement and an element that appears in weight loss products. Its anti-obesity activity is associated with high content of EA, equal to 10–100 µg EA/mL in the fruit extract, which, as demonstrated, has an influence on reducing the release of resisitin [59].

Studies based on animal models confirm the health promoting activity of EA. As demonstrated by Wang et al. [60], EA impacts lipid metabolism regulation as well as promotes white adipose tissue browning in a rat model. Additionally, a reduction in serum resistin level and improvement of hepatic steatosis and serum lipid profile were observed. Significant activity of EA was also recorded towards the downregulation of adipogenic and lipogenic gene expression in an animal model. Complementary analyses showed the anti-obesity effects of ellagic acid, focusing on its thermogenic and lipolytic activities in C57BL/6 mice with high-fat diet-induced obesity [61]. Anti-obesity activity of EA was confirmed several times by studies based on volunteers. One such study involved a 20-week procedure with a placebo group and an ellagic acid group, with participants receiving 3 mg of EA per day. Detail analysis revealed that participants of ellagic acid group significantly reduced body mass index (BMI), triglycerides (TG), fat ratio and visceral fat [62].

All of the studies explicitly indicate the anti-obesity activity of EA along with the improvement of basic factors that are strictly associated with the world-wide problem. Regular intake of EA can reduce weight and maintain it as a preventive measure against overweight and obesity.

## 3. Bioavailability and Safety of Ellagic Acid

As mentioned in previous sections, ellagic acid is an interesting compound due to its lipophilic and hydrophilic structural components as well as its wide spectrum of biological activities. However, there are some its disadvantages linked with its poor water solubility (9.7 µg/mL), short plasma half-life and poor oral bioavailability [62]. Following oral intake, its bioavailability is limited, primarily due to its poor absorption in the gastrointestinal tract, particularly in the stomach and proximal small intestine. Once absorbed, it undergoes extensive first-pass metabolism with minimal enterohepatic recycling, leading to rapid clearance from the body [13]. As a result, its plasma half-life is short, and the concentrations reached in tissues are generally insufficient for therapeutic efficacy.

Studies performed on a rat model showed that EA oral absorption was approx. 0.2% following administration of the compound as black raspberry powder, corresponding to 75 mg/L of EA [63]. Low bioavailability was also observed in pharmacokinetic analysis based on human studies. In these cases, the absorption was below 1% when EA was administered in the form of pomegranate juice or freeze-dried black raspberry [63].

The significant pro-health biological activities are difficult to utilize in practice due to its weak bioavailability [64]. However, there are some possible solutions that could help to address this issue. The most important of these are based on using derivatives of the compound, revealing much better bioavailability, or using a new drug delivery system, which allows us to improve the disadvantages of EA. A promising way to improve bioavailability was proposed by Mady and Ibrahim [65], who presented a solution based on a cyclodextrin-based nanosponge. Studies based on an animal model revealed that this approach can improve the oral bioavailability.

An important feature of each compound is its potential to have a toxic effect on the organism. Similarly to other compounds and drugs, EA was evaluated in this direction several times [66]. Studies in rats showed that after EA administration, there were occasional changes in hematological and serum biochemical parameters—such as mean corpuscular volume (MCV), aspartate transaminase (AST), and alkaline phosphatase (ALP)—in both males and females. However, these changes were considered incidental and not related to the treatment. Histopathological examination identified isolated lesions in organs such as the lungs, heart, liver, and kidneys, but their occurrence was comparable to that seen in the control group [67].

## 4. Effect of Ellagic Acid on Liver Condition

The liver plays a central role in maintaining physiological homeostasis through its involvement in numerous critical functions, including the synthesis and metabolism of carbohydrates, proteins, lipids, and the biotransformation of toxic substances prior to their distribution to other vital organs [68]. Hepatitis and steatohepatitis represent a significant proportion of hepatic disorders observed in developing regions. In the absence of treatment, these conditions have the potential to progress to cirrhosis, with the subsequent development of hepatocellular carcinoma (HCC) being a distinct possibility. A plethora of etiological factors have been implicated in the onset and progression of liver disease, including environmental toxins, pharmacological agents, lifestyle-related behaviors, and prior viral infections. Of particular significance are the following risk factors: chronic alcohol consumption, exposure to aflatoxins, drug-induced hepatotoxicity, obesity, insulin resistance, diabetes mellitus, and infection with hepatitis B and C viruses [69]. These factors substantially contribute to the pathogenesis and advancement of hepatic pathology. Therefore, effective prevention and therapeutic strategies for liver disease and its progression to HCC must prioritize the identification and mitigation of these modifiable risk factors. Additionally, the therapeutic potential of agents with antioxidant, anti-inflammatory, pro-apoptotic, and anti-fibrotic properties warrants further investigation in this context [70]. Ellagic acid has numerous pharmacological properties, including antioxidant, anti-inflammatory, apoptosis-mediating and anti-fibrotic activities [71].

However, the precise mechanism by which ellagic acid exerts its action in the context of chronic liver disease remains to be fully elucidated. Research has indicated that this natural compound may have the potential to prevent or reduce toxicity in the liver by inhibiting NF-κB activation and NO generation, and by enhancing the cellular antioxidant system. As demonstrated by García-Niño and Zazueta [12], research has shown the effectiveness of ellagic acid in protecting the liver against toxins such as ethanol, cyclosporine, rifampicin, cisplatin, isoniazid, mercury, paracetamol and CCl_4_. These compounds have been shown to impact liver function and structure.

Furthermore, the authors described molecular mechanisms including the following: free radical scavenging, regulation of cytokine production, synthesis of phase I and II enzymes, lipid synthesis and degradation processes and maintenance of oligoelement levels.

**Figure 4:**
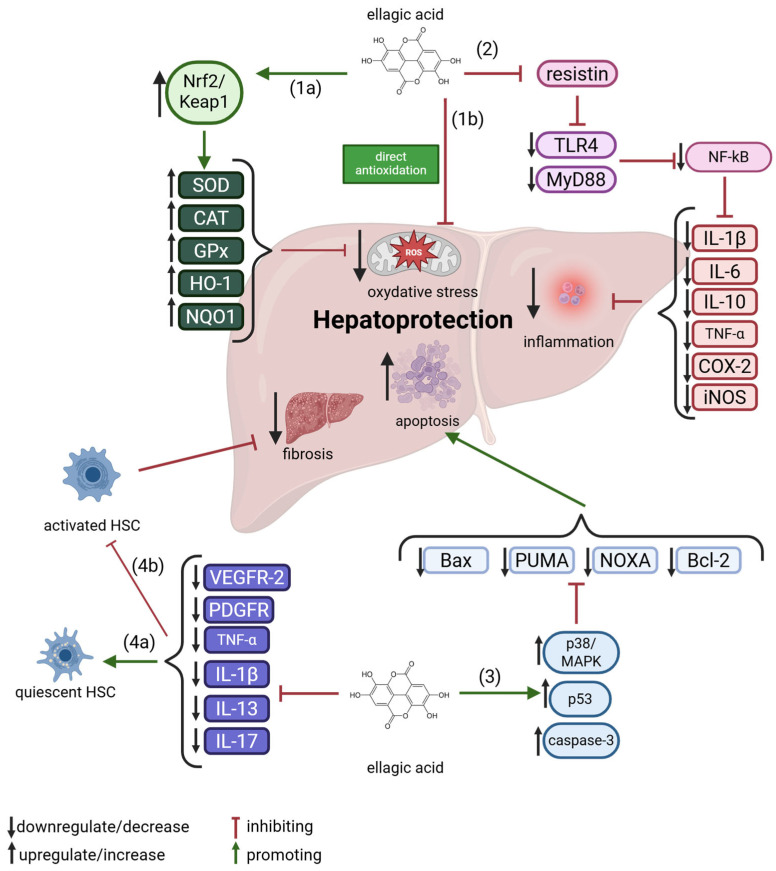
An overview of ellagic acid molecular mechanisms in hepatoprotection. 1a—indirect antioxidation, 1b—direct antioxidation, 2—anti-inflammation, 3—pro-apoptosis, 4a—promotion of quiescent of HSC, 4b—inhibition of activated HSC. For further explanations, please see text below. Abbreviations: Bcl-2—B-cell Lymphoma 2, CAT—Catalase, COX-2—Cyclooxygenase-2, GPx—Glutathione Peroxidase, HO-1—Heme Oxygenase-1, HSC—Hepatic Stellate Cell, IL—Interleukin, iNOS—Inducible Nitric Oxide Synthase, MAPK—Mitogen-Activated Protein Kinase, NF-κB—Nuclear Factor kappa-light-chain-enhancer of activated B cells, NOXA—Phorbol-12-Myristate-13-Acetate-Induced Protein 1, NQO1—NAD(P)H Quinone Dehydrogenase 1, Nrf2—Nuclear Factor Erythroid 2–Related Factor 2, PDGFR—Platelet-Derived Growth Factor Receptor, PUMA—p53 Upregulated Modulator of Apoptosis, ROS—Reactive Oxygen Species, SOD—Superoxide Dismutase, TLR4—Toll-Like Receptor 4, TNF-α—Tumor Necrosis Factor-alpha, VEGFR-2—Vascular Endothelial Growth Factor Receptor 2. Created with BioRender^®^.

Keshtzar et al. [72] reported that ellagic acid exhibits a protective effect against arsenic-induced hepatotoxicity. Arsenic, a pro-oxidant heavy metal and one of the most hazardous environmental toxins, exerts significant oxidative damage on hepatic tissue. The study demonstrated that ellagic acid, a naturally occurring phenolic compound, was capable of reversing reactive oxygen species (ROS) generation and preserving mitochondrial membrane integrity. It mitigated arsenic toxicity through both direct antioxidant activity and indirect mechanisms, such as the preservation of mitochondrial complex II function [5]. In a separate investigation, an in situ intestinal perfusion model was employed to assess the influence of ellagic acid on the oral bioavailability of metoprolol. The findings indicated that ellagic acid significantly enhanced metoprolol bioavailability by inhibiting the activity of cytochrome P450 2D6 (CYP2D6), an isoenzyme involved in the metabolism of approximately 25% of clinically used drugs. These results suggest a potential for herb–drug interactions when ellagic acid-containing supplements are co-administered with medications that are CYP2D6 substrates [73]. A schematic representation of molecular mechanisms underlying EA’s potential in hepatoprotection is shown in Figure 4 and comprehensively discussed in the subsequent sections.

Research has demonstrated that ellagic acid exhibits a markedly stronger protective effect against oxidative stress than vitamin E [29]. It has been estimated that EA interacts with hydroperoxyl radicals in aqueous environments at nearly twice the rate of Trolox. In support of this, a study revealed that blackberry extract—rich in ellagic acid—displayed superior antioxidant activity (IC_50_ = 20.3 ± 4.2 μg/mL) in comparison to Trolox. This extract effectively suppressed superoxide generation by NADPH oxidase in both THP1 monocytes and J744A.1 mouse ascites macrophage cell lines, suggesting substantial antioxidant potential. In vitro investigations further elucidated the mechanism by which ellagic acid mitigates oxidative stress and insulin resistance. Specifically, its action involves the activation of the Keap1-Nrf2 signaling pathway in HepG2 cells via the upregulation of miR-223. Under oxidative stress conditions, ellagic acid was shown to lower ROS and MDA concentrations while enhancing SOD activity in high-glucose-treated HepG2 cells [74]. Similarly, pomegranate seed extract at a concentration of 100 μg/mL completely reversed oxidative stress induced by tert-butyl hydroperoxide in HepG2 cells, attributable to its robust free radical scavenging capacity [75]. Additional studies on human hepatic cell lines indicated that treatment with ellagic acid (10 μM for 18 h) significantly attenuated oxidative stress [35]. The compound also demonstrated hepatoprotective properties by counteracting oxidative stress-induced hepatotoxicity. Specifically, ellagic acid prevented the uptake of ROS induced by vitamin K3 (VK3) and reduced apoptosis associated with mitochondrial depolarization—one of the primary sources of intracellular ROS. Furthermore, ellagic acid was effective in suppressing lipid peroxidation through its neutralizing activity against hydroxyl, peroxyl, and superoxide radicals [76].

Recent in vivo findings indicated that oral administration of ellagic acid (60 mg/kg/day) to Sprague-Dawley rats significantly reduced lipid peroxidation, thereby preserving cell membrane integrity and restoring normal hepatic histoarchitecture [77]. At a lower dosage (30 mg/kg), ellagic acid also inhibited hepatic cytochrome P450 enzymes, suggesting its potential to mitigate alcohol-induced liver injury. Additional in vivo studies showed that ellagic acid protects against concanavalin A-induced hepatic damage by lowering liver enzyme levels and total bilirubin concentration [78]. In a systematic, randomized controlled trial conducted by Block et al., involving 2446 participants, the efficacy of 24 antioxidants was evaluated in mitigating dose-dependent drug toxicity. Among the compounds studied—including glutathione, vitamin A, N-acetylcysteine, melatonin, vitamin E, selenium, coenzyme Q10, L-carnitine, and ellagic acid—ellagic acid exhibited the most potent antioxidant activity. Notably, it demonstrated the highest efficacy in reducing chemotherapeutic drug toxicity when used as an adjuvant [79].

### 4.1. Inhibition of Hepatic Inflammation

A substantial body of evidence supports a strong association between oxidative stress and inflammation [80]. One of the principal transcription factors involved in pro-inflammatory signaling is nuclear factor kappa B (NF-κB), which is ubiquitously expressed across cell types and becomes activated in response to cellular stressors [81]. Although the precise molecular mechanism by which ellagic acid modulates pro-inflammatory cytokine activity remains to be fully elucidated, it is hypothesized that this polyphenolic compound exerts its anti-inflammatory effects primarily through direct inhibition of the NF-κB signaling pathway. For instance, Ahada et al. [82] demonstrated that ellagic acid ameliorated dyslipidemia and attenuated diabetic nephropathy in type 2 diabetic Wistar rats, an effect attributed to the suppression of NF-κB pathway expression. NF-κB is also critically involved in regulating the transcription of cyclooxygenase-2 (COX-2), an enzyme essential to the inflammatory response [27]. Administration of ellagic acid at a dose of 100 mg/kg body weight has been shown to downregulate COX-2 mRNA expression, primarily by reducing reactive oxygen species (ROS) production, thereby indirectly inhibiting NF-κB activation [11].

Multiple pro-inflammatory cytokines are central to the orchestration of immune responses. The macrophage migration inhibitory factor (MIF) has been identified as a potent inducer of NF-κB activation and cellular chemotaxis during inflammatory processes [2]. Ellagic acid, at a concentration of 50 μM, was found to suppress MIF’s tautomerase activity and reduce MIF-mediated pro-inflammatory signaling in peripheral blood mononuclear cells [43]. Furthermore, ellagic acid inhibited the expression of key inflammatory mediators, including TNF-α, IL-6, and the C-C motif chemokine, in lipopolysaccharide (LPS)-stimulated macrophages and adipocytes, indicating its potential to reduce inflammation in adipose tissue [83]. Even at relatively low concentrations (6.25 μM and 12.5 μM), ellagic acid significantly decreased TNF-α and IL-6 levels in LPS-stimulated RAW264.7 macrophage cells [84].

Notably, ellagic acid has also been shown to affect resistin—a pro-inflammatory adipokine implicated in linking obesity to type 2 diabetes [85]. Pomegranate fruit extract enriched with ellagic acid was found to suppress elevated serum resistin concentrations in murine models. In vitro studies further revealed that ellagic acid lowered resistin levels in 3T3-L1 adipocytes. In a subsequent study, the same research group observed that ellagic acid reduced circulating resistin levels and hepatic lipid accumulation in KK-Ay mice, despite having no effect on resistin mRNA expression in adipose tissue [86].

The pro-inflammatory response initiated at sites of hepatic injury involves the release of cytokines such as tumor necrosis factor-α (TNF-α), interleukin-6 (IL-6), and interleukin-1β (IL-1β)—along with the recruitment of immune cells, including CD8^+^ T lymphocytes, Th17 cells, and B cells [87]. This immune activation has been shown to drive excessive cellular proliferation, which contributes to increased reactive oxygen species (ROS) production and subsequent DNA damage, thereby fostering the accumulation of somatic mutations. When this elevated proliferative activity coincides with genomic instability, it can facilitate oncogenic transformation. Furthermore, persistent inflammation has been shown to alter the hepatic immune microenvironment, potentially allowing malignant cells to escape immune surveillance mechanisms [88].

Ellagic acid and its metabolite, Urolithin A (URO A), have demonstrated immunomodulatory effects in lipopolysaccharide (LPS)-stimulated U937 and THP-1 monocyte cell lines. Both compounds significantly reduced the expression of NF-κB and interleukin-10 (IL-10) following LPS exposure. Additionally, ellagic acid suppressed toll-like receptor 4 (TLR-4) expression, suggesting that its immunomodulatory and anti-inflammatory properties may be mediated through both TLR-4/NF-κB-dependent and -independent mechanisms [89]. In another investigation, the anti-inflammatory activity of ellagic acid was evaluated using RAW264.7 macrophages pretreated with varying concentrations of the compound (1, 10, and 50 μM) prior to LPS stimulation (1 μg/mL). The findings indicated that ellagic acid exerted a concentration-dependent anti-inflammatory effect primarily via the TLR-4/NF-κB signaling axis. Specifically, ellagic acid inhibited the LPS-induced phosphorylation and degradation of IκB, as well as the nuclear translocation of the NF-κB subunit p65, thereby suppressing downstream inflammatory responses [90]. The anti-inflammatory efficacy of ellagic acid (30 mg/kg body weight) was also confirmed in a rat model exposed to acrylamide (20 mg/kg body weight), wherein the compound significantly attenuated inflammatory cytokine expression (TNF-α, IL-6, and IL-1β) [91].

Similarly, in a murine model of acute liver injury induced by LPS and D-galactosamine, ellagic acid was found to inhibit NF-κB activation, thereby regulating the inflammatory activities of neutrophils and macrophages at the site of hepatic injury. In addition to its anti-inflammatory effects, ellagic acid also modulated the antioxidant response by promoting nuclear factor erythroid 2-related factor 2 (Nrf2) activation and upregulating heme oxygenase-1 (HO-1) expression, ultimately protecting liver tissue from oxidative damage [92].

Another study investigated the impact of pomegranate juice, containing 40% ellagic acid, on rats fed a high-sugar, high-fat diet and diagnosed with non-alcoholic fatty liver disease. Administration of 60 mL of the juice resulted in decreased hepatic expression of pro-inflammatory cytokines (TNF-α, IL-6, IL-1β) and transforming growth factor-β (TGF-β), thereby reducing liver inflammation [93].

Moreover, ellagic acid derived from raspberries was shown to alleviate metabolic disturbances in both HepG2 cells (treated with 100–300 μg/mL) and male C57BL/6 mice (fed a 0.03% supplemented diet), primarily through mitigation of hepatic oxidative stress [13]. As demonstrated by both in vitro and in vivo studies, a reduction in metabolic complications has been shown to be achieved by means of the alleviation of oxidative stress in the liver.

The present findings suggest that ellagic acid may have the potential to normalize metabolic disorders caused by high sugar and fat intake. It is regrettable that clinical reports on the anti-inflammatory effects of ellagic acid in the human liver are not available. Therefore, it is recommended that extensive clinical trials be conducted.

### 4.2. Apoptosis Mediating Effect of EA

Mitochondria-derived intracellular signaling has been demonstrated to play a pivotal role in the precise regulation of apoptosis, the mechanism of programmed cell death. Concurrently, the initiation of the extrinsic apoptosis pathway is triggered by the activation of cell surface receptors containing the death domain. In vitro studies have indicated that ellagic acid displays anticancer properties by inducing cell cycle arrest at the G2/M stage and enhancing apoptotic mechanisms in cancer cells [94]. It is important to note that the ellagic acid metabolite, URO A (IC50 = 200 μM), increased the expression of the tumor suppressor protein p53 and its proapoptotic effectors in HepG2 cell lines threefold: Bax, PUMA and NOXA—3-, 3- and 7-fold, respectively [95]. In the same study, the expression of the activated form of caspase-3 and phosphorylated p38 MAPK increased by 1.3- and 1.7-fold, respectively. Furthermore, URO A significantly decreased the phosphorylation level of c-Jun protein, indicating its role in the modulation of the p38-MAPK pathway and interaction with p53, which is important in the process of metastasis and elimination of cancer cells.

In the context of in vivo studies, the hepatoprotective effects of ellagic acid were evaluated in a model of apoptosis induced by the cytotoxic drug methotrexate [96]. The administration of ellagic acid (5 and 10 mg/kg) orally, following a single intraperitoneal injection of methotrexate (20 mg/kg), exhibited a protective effect against mitochondrial function and apoptosis in liver tissue, as evidenced by a 10-day observation period. This effect was associated with activation of the transcription factor Nrf2 and inhibition of the NF-κB signaling pathway, suggesting a potential therapeutic application of ellagic acid in reducing drug-induced hepatotoxicity. Analogous effects were observed in the carbon tetrachloride-induced hepatic toxicity model (CCl_4_), where the administration of ellagic acid (10 mg/kg, i.p.) resulted in activation of caspase-3 and suppression of Bcl-2 protein expression [97]. The results of this study suggest that ellagic acid has the capacity to induce apoptosis via both the mitochondrial pathway and independently of it.

A study utilizing a mouse model of iron overload-induced toxicity demonstrated that ellagic acid (2–8 mg/kg, i.p.), extracted from *Clerodendrum viscosum*, diminished oxidative stress and augmented the expression of proteins implicated in apoptosis, such as caspase-3 and PARP, thereby substantiating its therapeutic potential in instances of iron accumulation-related disorders [98]. Protein kinase C (PKC) isotypes, which have been identified as key regulators of cell proliferation, differentiation and death, have been identified as molecular targets of ellagic acid. In a mouse model of hepatic lymphoma, a daily dose of ellagic acid (40–80 mg/kg for 15 days) led to a significant decrease in tumor volume and ascites cell proliferation, alongside an increase in caspase-3 activity [99]. However, it should be noted that to date, there is an absence of clinical evidence to support the proapoptotic effect of ellagic acid in human liver disease.

### 4.3. Anti-Fibrotic Effect of EA

The initiation of hepatic fibrogenesis is closely linked to a cascade of inflammatory responses involving various cellular components, such as platelets, macrophages, and sinusoidal endothelial cells, as well as soluble signaling molecules including transforming growth factor-β (TGF-β), platelet-derived growth factor-BB (PDGF-BB), and cytokines such as IL-1β, IL-13, and IL-17. These elements collectively contribute to liver repair and regenerative responses by activating innate immune pathways. A central inflammatory mechanism driving fibrotic transformation in the liver is the activation of hepatic stellate cells (HSCs)—a specific pericyte population within the liver—as well as portal tract myofibroblasts [100]. HSCs are situated within the perisinusoidal space, also referred to as the space of Disse, positioned between hepatic endothelial cells and hepatocytes. Upon activation, these cells undergo a phenotypic transformation from a quiescent to a migratory and fibrogenic state, acquiring the ability to secrete extracellular matrix (ECM) components such as collagens (particularly types I and III), fibronectin, laminin, and proteoglycans in response to hepatic injury. Myofibroblasts—largely derived from activated HSCs—exhibit properties of both fibroblasts and smooth muscle cells, enabling them to contribute structurally and functionally to fibrosis development.

This activation process is mediated by pro-inflammatory cytokines including IL-1β, IL-13, and IL-17, and is further perpetuated by TGF-β signaling, which plays a pivotal role in advancing fibrotic pathology [101]. Once activated, HSCs interact dynamically with hepatocytes, Kupffer cells, macrophages, and endothelial cells to orchestrate a localized pro-fibrotic microenvironment, primarily through ECM protein deposition. The progression of fibrosis results in the excessive accumulation of collagens I and III, fibronectin, laminin, and proteoglycans, replacing the normal type IV collagen predominantly produced by quiescent HSCs in healthy livers [102]. The fibrogenic response is further amplified by continuous expression of growth factors such as PDGF-BB, TGF-β, and vascular endothelial growth factor (VEGF) from both activated HSCs and differentiated myofibroblasts. Notably, there is evidence suggesting that the reversion of activated HSCs to their quiescent phenotype is possible if inflammatory stimuli and cytokine levels are sufficiently suppressed, offering a potential therapeutic window for halting fibrogenesis [101]. Thus, the interplay among inflammatory mediators, effector cells (HSCs and myofibroblasts), and ECM regulatory signals constitutes a compelling target for antifibrotic strategies.

Ellagic acid has demonstrated the capacity to disrupt this pathological triad by modulating inflammatory cytokines, inhibiting activation of fibrogenic cells, and limiting ECM protein production. In endothelial tumor cell lines (U87 and HT1080), EA significantly impeded the phosphorylation of PDGFR and VEGFR-2, effectively downregulating ERK 1/2 signaling pathways implicated in cell survival [94]. Earlier investigations have established that IL-1β, IL-13, and IL-17 potentiate HSC activation and proliferation via JNK-mediated phosphorylation of Smad-2/3, further entrenching hepatic fibrogenesis. Ellagic acid mitigates this process by downregulating Smad 2/3 phosphorylation through modulation of cytokine levels at the site of injury, thereby obstructing fibrosis progression [103].

The TGF-β signaling axis represents a particularly viable target for pharmacological intervention, with numerous studies affirming its centrality in regulating HSC activation and inflammatory cascades [102]. Ellagic acid exerts its therapeutic potential by attenuating this pathway, thereby inhibiting ECM accumulation and offering promising antifibrotic effects [5]. Moreover, oxidative stress and ROS generated by Kupffer cells, monocytes, and macrophages further contribute to hepatocellular damage and HSC activation. In vitro studies have demonstrated that ellagic acid, at a concentration of 6 μg/mL, exhibits significant antioxidant activity, scavenging ROS and exerting hepatoprotective effects. This antioxidative mechanism helps prevent hepatocyte injury and subsequent fibrotic scarring [101]. Consequently, ellagic acid and its analogues present a strong therapeutic prospect in the treatment of liver fibrosis, potentially arresting its progression toward cirrhosis and hepatocellular carcinoma.

The potential of ellagic acid for the treatment of liver diseases—in vitro studies and clinical trials—is shown in Table 2.

### 4.4. Modulation of Gut Microbiota

Ellagic acid has poor bioavailability when administered orally, mainly due to its low water and body fluid solubility, and low lipophilicity [114]. Interestingly, EA can be metabolized in the gut by certain microorganisms to form urolithins (UROs), the main derivatives, which can pass through colonocytes and be absorbed and distributed to target tissues [115]. Urolithins are a large group of ellagic acid metabolites, formed by opening the lactone rings and subsequent decarboxylation and dihydroxylation in various positions. These reactions are catalyzed by gut microbiota enzymes and ultimately form two main URO subtypes: urolithin A (URO A) and urolithin B (URO B), as well as their structural isomers (i.e., isourolithin A and isourolithin B). Certain bacterial species have been documented to express essential enzymes such as lactonase, decarboxylase and pyrocatechol-dehydroxylase, which are involved in the biotransformation of ellagic acid to urolithins. The most widely studied microorganisms are *Gordonibacter urolithinfaciens*, *Ellagibacter isourolithinifaciens*, *Enterocloster bolteae* and *Enterococcus faecium*, among others [115,116]. Furthermore, studies have shown that individuals respond differently to the administration of ellagic acid and its precursors, measured by urolithin production. As different urolithin types can exhibit diverse biological activity, three main URO metabolism phenotypes have been proposed: UM-A, which produces mainly URO A; UM-B, which synthesizes more iso URO A and URO B and less URO A; and URO-0, which has no measurable content of the aforementioned urolithins [117]. Studies have shown that a higher prevalence of UM-B is found in patients with metabolic syndrome spectrum disorders (e.g., obesity and cardiovascular diseases) than in healthy subjects [115]. This suggests that there may be a link between the composition of the gut microbiota and the beneficial effects of EA derivatives. The gut microbiota plays an important role in the metabolic pathways of EA and the further production of UROs, and the composition and function of the intestinal microbiome can also be modulated by EA and its derivatives. Numerous studies have reported the beneficial effects of EA on gut microbiota. Zhang et al. [118] used an alcohol-related liver disease (ALD) mouse model to examine the potential of URO-A to alleviate ALD symptoms. It was confirmed that the administration of URO-A significantly altered the composition of the gut microbiota, particularly by increasing the abundance of *Bacteroides sartorii*, *Parabacteroides distasonis*, and *Akkermansia muciniphila*, which can produce propionate—one of the most important short-chain fatty acids (SCFAs) for maintaining proper gut function [118]. The study by Luo et al. evaluated the beneficial effects of EA in a mouse model of a high-fructose diet. It has been suggested that excessive fructose intake results in changes to the composition of the gut microbiota, which may lead to liver diseases such as MASLD. The study reported that intragastric administration of EA significantly decreased the levels of Colidextribacter, Ruminococcus and Alislipes, which were previously promoted by high fructose intake and are associated with liver disease. Notably, the content of the beneficial Faecalibacterium, which has the ability to produce SCFAs, increased [119]. Appropriate production of SCFAs (i.e., acetate, propionate and butyrate) and maintaining their ratio (3:1:1, respectively) is essential for lipid and carbohydrate metabolism, reducing inflammatory processes, and regulating the growth of beneficial microorganisms in the gut while decreasing the level of detrimental species [120,121]. The modulation of intestinal microbiota composition may be a very important feature of EA and its derivatives in the context of liver disease prevention and treatment. A comprehensive review of the role of EA and UROs in the gut microbiome has been recently published elsewhere [122]. In the following section, however, we discuss a specific role of EA in liver steatosis.

## 5. Role of Ellagic Acid in MASLD

Metabolic dysfunction-associated steatotic liver disease (MASLD) is a chronic and multifaceted disorder, involving two types of conditions—simple steatosis and non-alcoholic steatohepatitis [123]. MASLD is characterized by excessive accumulation of lipids, mainly in the form of triglycerides in the hepatocytes. Progression of the disease results in fibrosis, cirrhosis and ultimately hepatocellular carcinoma [124]. Simple steatosis is a fully reversible condition and, for this reason, is frequently considered a benign disease, though its progression leads to significantly severe complications, even to death [125,126,127]. Global prevalence of MASLD is estimated at 32% among adults globally [128] and it is constantly increasing [129], which makes it a serious global burden. Until recently, a spectrum of liver steatosis was named non-alcoholic fatty liver disease (NAFLD); however, in 2023 it was proposed to rename it to MASLD in order to avoid potentially stigmatizing language and underline the much more complex cause–effect relationship underlying these disorders [130]. The pathophysiology of MASLD is complex, multipronged and, despite significant advances in recent years, still not fully understood. Currently, the most comprehensive theory of MASLD pathogenesis is the ‘parallel multiple-hit’ hypothesis [131], in which such processes as inflammation, oxidative stress, disruption in carbohydrate and lipid metabolism, insulin resistance, autophagy and alterations in gut microbiota composition as well as genetic factors contribute to liver steatosis [132]. MASLD is often perceived as a hepatic manifestation of metabolic syndrome (MetS). It remains unclear whether diseases included in MetS are directly related to MASLD development or if the relationship between these two spectra is bidirectional. In other words, the question of whether the presence of MALSD could be a predictor of other metabolic diseases, e.g., hypertension, diabetes, obesity or dyslipidemia, needs to be elucidated [133]. Despite the fact that there are a number of studies concerning EA or urolithin efficacy in oxidative stress, inflammatory processes or gut microbiota modulation, only limited data is available on MASLD. One of the most interesting features of EA’s potential in MASLD is the impact on the gut–liver axis and reducing gut permeability. In the study by Kim et al. [134], 50 mg/kg of EA was administered daily to high-fat diet-induced MASLD mice. A significant reduction in gut permeability and circulating endotoxins, mainly lipopolysaccharide (LPS), was observed, resulting in the suppression of hepatic inflammation and fibrosis [134]. Also, in the MASLD mouse model, Ren et al. administered defatted walnut powder extract, rich in ellagic acid. The study shows significant changes in gut microbiota composition. A reduction in Erysipelotrichia, Firmicutes and Actinobacteria content and increased abundance of Bacterioidedes, Clostridiales and Prevotellaceae were reported. Studies have shown that Prevotellaceae and Bacterioidetes are involved in SCFA synthesis in the gut. SCFAs in turn could inhibit de novo lipogenesis, normalize lipid profile and reduce hepatic steatosis and inflammation [135]. What is more, certain EA metabolites, namely urolithin C, may also be beneficial in maintaining proper gut microbiota composition, therefore alleviating and preventing MASLD. In the study by Xu et al. [136], a mouse model of choline-deficient amino acid-defined high-fat diet (CDAHFD)-induced MASLD was used and URO C was efficient as a preventive agent, as well as in maintaining the proper ratio of Firmicutes/Bacterioides in the gut [136]. It is well-documented that dysbiosis contributes to MASLD development by increasing gut permeability and inducing hepatic inflammation, resulting in steatohepatitis and fibrosis [137]. To sum up, restoring and rebalancing the composition of the gut microbiota may become one of the most important strategies in MASLD prevention and treatment. On the other hand, EA and its microbial derivatives also have antioxidant and anti-inflammatory potential via inhibiting de novo lipogenesis and decreasing levels of fatty acid synthase, SERBP-1c, and xanthine oxidase, as reported in the study by Elsewidy et al. [138]. The study showed that ellagic acid can be more effective than the commonly used drug allopurinol in alleviating hyperuricemia and related MASLD in the high-fat diet albino rat model [138]. Based on promising in vivo experiments, a randomized double-blind clinical trial investigating the effects of EA in patients with MASLD has been designed by Mighani et al. [139]. Forty-four recruited patients were randomized and divided into two equinumerous groups: the studied group, which received 180 mg of EA per day, and the control group, which received a placebo for 8 weeks. At the end of the study, the intervention group had significantly reduced insulin resistance, TG and LDL as well as liver aminotransferases AST, ALT, ALP, GGT and ultimately serum level of C-reactive protein. Also, an increase in the mean total antioxidant capacity (TAC) was observed. However, there were no significant differences in total cholesterol, HDL or fasting blood sugar levels between the groups [139]. The short duration (8 weeks) and small sample size (44 subjects) were the most important limitations, which may influence the end results. Although promising results have been reported, further studies are necessary to fully elucidate the potential of ellagic acid in MASLD.

## 6. Conclusions

It has been demonstrated that ellagic acid exhibits a broad spectrum of biological activity, with its hepatoprotective properties being of particular importance. The protective effects of EA on the liver are primarily attributable to its strong antioxidant potential, which is based on its ability to directly scavenge oxygen free radicals and chelate transition metal ions that catalyze free radical reactions. Consequently, EA has been demonstrated to effectively mitigate oxidative stress, a pivotal pathogenic factor in hepatocyte damage, including fatty liver disease, inflammation, and fibrosis.

Furthermore, EA has the capacity to modulate the inflammatory response by inhibiting the production of pro-inflammatory cytokines (e.g., TNF-α, IL-1β, IL-6) and reducing the activation of the nuclear transcription factor NF-κB. Concurrently, it impacts the activity of enzymes implicated in the biotransformation of xenobiotics—encompassing both phase I (e.g., cytochromes P450) and phase II (e.g., glutathione transferases, sulfotransferases)—thereby contributing to the detoxification of hepatotoxic metabolites and the preservation of hepatic cells. EA also regulates lipid metabolism by influencing the expression of genes involved in lipogenesis (SREBP-1c, FASN) and β-oxidation of fatty acids (PPARα, CPT1), which may be important in the prevention of MASLD.

Furthermore, EA has been demonstrated to exhibit multidirectional molecular mechanisms involving both antioxidant properties and modulation of signaling pathways relevant to the survival, proliferation, and invasiveness of cancer cells. EA has been documented as an inducer of programmed cell death (apoptosis) through the activation of the mitochondrial pathway. This process is associated with increased expression of proapoptotic proteins, including Bax, cytochrome c, and caspase-3, while concomitantly inhibiting the expression of the antiapoptotic protein Bcl-2. EA has also been demonstrated to inhibit the activation of key signaling kinases, such as the phosphorylated form of STAT3, Akt kinase, and ERK1/2, thereby leading to the inhibition of the PI3K/Akt/mTOR pathway, which is often overactive in cancer cells. Furthermore, a reduction in cyclin D1 expression has been observed, resulting in cell cycle arrest in the G1 phase and reduced cell proliferation. It is evident that EA exerts a significant influence on various processes associated with tumor progression. A notable example of this is its capacity to inhibit the expression of vascular endothelial growth factor (VEGF), thereby regulating angiogenesis. Additionally, EA has been observed to impede the progression of invasion and metastasis by constraining the activity of matrix metalloproteinases (MMPs), specifically MMP-2 and MMP-9, which play a crucial role in the process of metastasis. It has been demonstrated that EA exerts a significant capacity to curtail the extent of DNA damage instigated by genotoxic agents, both in a direct capacity and by modulating the function of DNA repair enzymes.

Despite its clear therapeutic potential, the clinical use of EA is significantly limited by its unfavorable pharmacokinetic properties, in particular its very low solubility in aqueous environments and limited permeability of biological membranes. Consequently, this results in suboptimal gastrointestinal absorption, accelerated clearance, and diminished bioavailability following oral administration. In response to these challenges, a number of innovative active substance delivery systems have been developed, including phospholipid complexes (phytosomes), thermosensitive liposomes, polymeric nanoparticles (e.g., PLGA), nanostructured lipid carriers (NLCs), and metal–organic frameworks (MOFs). Each of these strategies has been designed to increase the chemical stability of EA, prolong its circulation time in the blood, and improve its bioavailability in vivo.

Notwithstanding the encouraging outcomes observed in a plethora of preclinical studies employing animal and cell models, the clinical evidence pertaining to the effectiveness and safety of EA in human subjects remains sparse. A significant proportion of extant studies are of a preliminary nature, frequently comprising small subject groups. The absence of standardized methodologies and the diversity of preparations utilized complicate the comparison of results and the drawing of clear clinical conclusions. Nevertheless, given the favorable safety profile of EA and the growing body of evidence pointing to its multidirectional biological activity, this compound remains the subject of intense interest as a candidate for a bioactive ingredient in functional foods, dietary supplements, and phytotherapeutics supporting the treatment of chronic diseases, including liver diseases.

## Figures and Tables

**Table 1:** Examples of sources of ellagic acid.

Source of EA	Amount/Comments	Ref.
*Acalypha hispida* Burm.f.	119.4 mg/100 g and 540.9 mg/100 g of dry ethanol and aqueous extracts, respectively	[18]
*Castanea crenata* Sieb. & Zucc.	1.74 mg/g extract and 2.26 mg/g leaves	[19]
*E. angustifolia* L.	8.52 mg/g in leaf water extract	[20]
*Eugenia uniflora* L.	0.2% (2.72 *w*/*w*)—crude extract; 0.035% (3.90 *w*/*w*)—aqua fractions; 0.323% (4.05 *w*/*w*)—ethyl acetate fraction	[21]
*Myrtus communis* L.	3.88 mg/g leaf dry weight	[22]
*Pandiaka angustifolia* (Vahl) Hepper	65.44% in leaves	[23]
*Phyllanthus amarus* Schum. & Thonn. (Euphorbiaceae)	2.06% determined in extract using HPLC method	[24]
*Salacia chinensis* L.	51.60 mg/g of dry weight	[25]
*Syzygium calophyllifolium* (Wight) Walp.	88% of TPC analyzed in fruits	[26]

**Table 2:** Potential of ellagic acid against liver diseases—in vitro studies and clinical trials.

Type of Study	Cell Line/Model	Dose	Results	Ref.
In vivo	BALB/c mice	50–200 mg/kg of body weight	↓ plasma aminotransferase ↓ liver necrosis ↓ levels of TLR2 and TLR4 mRNA in liver ↓ NF-κB in liver ↓ IκB-α degradation levels in liver ↓ TNF-α, IL-6 and IL-1β	[104]
In vivo	Wistar rats	0.8 g/kg food	↓ NF-κB in liver ↑ Nrf2 i CPT1 in liver	[105]
In vivo	Nicotinamide induced diabetic rats	20 mg/kg BW	improvement in liver function markers ↓ hyperglycemia ↓ dyslipidemia	[106]
In vivo	BALB/c mice	2 g/100 g diet	↑ plasma insulin and ↓ plasma glucose levels ↓ triglyceride in plasma	[107]
In vivo	FVB/N mice HepG2	150 or 300 mg/kg BW 0–40 μM	↓ SREBP-1/FASN axis ↓ AKT-triggered hepatic de novo lipogenesis,	[108]
In vivo and in vitro	Sprague-Dawley rats	60 mg/kg BW	↓valproic acid induced hepatic injury	[77]
	Mice	10 mg/kg BW	↓ cisplatin induced hepatotoxicity ↓ peroxidative damage to liver tissue	[109]
In vivo	Wistar rats	10 mg/kg BW	↓ CCl_4_ induced liver damage ↑ Nrf2 in liver ↓ NF-κB in liver	[97]
In vivo	Wistar rats	60 mg/kg BW	↓ AlCl_3_ induced hepatic function impairment, ↓ dyslipidemia and hepatic histological alterations ↓ MDA and PCC ↑ CAT, GPx and SOD activity in liver ↑ GSH	[8]
In vivo	Diabetic male mice	*Lagerstroemia speciosa* extracts (4 g of ellagic acid)	↓ blood glucose, body weight, body fat ↑ insulin	[110]
In vivo	Goto-Kakizaki female rats	50 mg/kg BW	↓ IR lipid accumulation and oxidative stress ↑ insulin signaling pathway in the liver	[111]
In vivo	Non-obese type 2 diabetic rats	50 mg/kg BW	↑ serum insulin, β-cell size, β-cells number ↓ liver TBARS and glucose intolerance in rats	[112]
Clinical trial	180 mg, for 8 weeks, p.o.	44 patients	↓ BS, IR, HbA1c, TC, TG, MDA and TNF-α ↑ TAC level and activity of GPx, SOD	[113]

↑ increase in level; ↓ decrease in level; explanations of other abbreviations can be found in the ‘Abbreviations’ section.

## Data Availability

Not applicable.

## Abbreviations

AKT	Protein Kinase B
ALD	Alcohol-Related Liver Disease
ALT	Alanine Aminotransferase
AMPK	AMP-Activated Protein Kinase
AST	Aspartate Aminotransferase
ATP	Adenosine Triphosphate
Bax	Bcl-2 Associated X Protein
Bcl-2	B-cell Lymphoma 2
BS	Blood Sugar
CAT	Catalase
CCl_4_	Carbon Tetrachloride
COX-2	Cyclooxygenase-2
CPT1	Carnitine Palmitoyltransferase 1
CYP2D6	Cytochrome P450 2D6
EA	Ellagic Acid
ECM	Extracellular Matrix
ERK1/2	Extracellular Signal-Regulated Kinases 1/2
FASN	Fatty Acid Synthase
GPx	Glutathione Peroxidase
GSH	Glutathione
HbA1c	Hemoglobin A1c
HO-1	Heme Oxygenase-1
HSC	Hepatic Stellate Cell
IC_50_	Half-Maximal Inhibitory Concentration
IL	Interleukin
IL-1β	Interleukin 1 Beta
IL-6	Interleukin 6
IκB-α	Inhibitor of NF-κB Alpha
iNOS	Inducible Nitric Oxide Synthase
IR	Insulin Resistance
JNK	c-Jun N-terminal Kinase
LPS	Lipopolysaccharide
MAPK	Mitogen-Activated Protein Kinase
MASLD	Metabolic Dysfunction-Associated Steatotic Liver Disease
MCV	Mean Corpuscular Volume
MDA	Malondialdehyde
MIF	Macrophage Migration Inhibitory Factor
NAFLD	Non-Alcoholic Fatty Liver Disease
NF-κB	Nuclear Factor kappa-light-chain-enhancer of activated B cells
NO	Nitric Oxide
NOXA	Phorbol-12-Myristate-13-Acetate-Induced Protein 1
NSAIDs	Non Steroidal Anti Inflammatory Drugs
NQO1	NAD(P)H Quinone Dehydrogenase 1
Nrf2	Nuclear Factor Erythroid 2–Related Factor 2
PCC	Protein Carbonyl Content
PD	Parkinson’s Disease
PDGFR	Platelet-Derived Growth Factor Receptor
PI3K	Phosphatidylinositol 3-Kinase
PUMA	p53 Upregulated Modulator of Apoptosis
ROS	Reactive Oxygen Species
SCFAs	Short-Chain Fatty Acids
Smad2/3	Mothers Against Decapentaplegic Homolog 2/3
SOD	Superoxide Dismutase
SREBP-1	Sterol Regulatory Element Binding Protein 1
STAT3	Signal Transducer and Activator of Transcription 3
TAC	Total Antioxidant Capacity
TBARS	Thiobarbituric Acid Reactive Substances
TC	Total Cholesterol
TG	Triglycerides
TGF-β	Transforming Growth Factor Beta
TLR2	Toll-Like Receptor 2
TLR4	Toll-Like Receptor 4
TNF-α	Tumor Necrosis Factor-alpha
URO/URO-A/URO-B/URO-C	Urolithins A, B, C—gut microbial metabolites of EA
UM-A/UM-B/URO-0	Urolithin Metabotypes A, B, and Non-producer
VEGFR-2	Vascular Endothelial Growth Factor Receptor 2

## References

[1] Shakeri A., Zirak M.R., Sahebkar A. (2018). Ellagic Acid: A Logical Lead for Drug Development?. Curr. Pharm. Des..

[2] Ríos J.-L., Giner R.M., Marín M., Recio M.C. (2018). A Pharmacological Update of Ellagic Acid. Planta Medica.

[3] Bedel H.A., Usta C. (2025). Effect of Ellagic Acid on BDNF/PI3K/AKT-Mediated Signaling Pathways in Mouse Models of Depression. Iran. J. Basic. Med. Sci..

[4] Galano A., Francisco Marquez M., Pérez-González A. (2014). Ellagic Acid: An Unusually Versatile Protector against Oxidative Stress. Chem. Res. Toxicol..

[5] Baradaran Rahimi V., Ghadiri M., Ramezani M., Askari V.R. (2020). Antiinflammatory and Anti-Cancer Activities of Pomegranate and Its Constituent, Ellagic Acid: Evidence from Cellular, Animal, and Clinical Studies. Phytother. Res..

[6] Gupta A., Singh A.K., Kumar R., Jamieson S., Pandey A.K., Bishayee A. (2021). Neuroprotective Potential of Ellagic Acid: A Critical Review. Adv. Nutr..

[7] Tiwari M.K., Mishra P.C. (2013). Modeling the Scavenging Activity of Ellagic Acid and Its Methyl Derivatives towards Hydroxyl, Methoxy, and Nitrogen Dioxide Radicals. J. Mol. Model..

[8] Salem A.M., Mohammaden T.F., Ali M.A.M., Mohamed E.A., Hasan H.F. (2016). Ellagic and Ferulic Acids Alleviate Gamma Radiation and Aluminium Chloride-Induced Oxidative Damage. Life Sci..

[9] Kilic I., Yeşiloğlu Y., Bayrak Y. (2014). Spectroscopic Studies on the Antioxidant Activity of Ellagic Acid. Spectrochim. Acta Part. A Mol. Biomol. Spectrosc..

[10] de Oliveira M.R. (2016). The Effects of Ellagic Acid upon Brain Cells: A Mechanistic View and Future Directions. Neurochem. Res..

[11] El-Shitany N.A., El-Bastawissy E.A., El-desoky K. (2014). Ellagic Acid Protects against Carrageenan-Induced Acute Inflammation through Inhibition of Nuclear Factor Kappa B, Inducible Cyclooxygenase and Proinflammatory Cytokines and Enhancement of Interleukin-10 via an Antioxidant Mechanism. Int. Immunopharmacol..

[12] García-Niño W.R., Zazueta C. (2015). Ellagic Acid: Pharmacological Activities and Molecular Mechanisms Involved in Liver Protection. Pharmacol. Res..

[13] Kang I., Buckner T., Shay N.F., Gu L., Chung S. (2016). Improvements in Metabolic Health with Consumption of Ellagic Acid and Subsequent Conversion into Urolithins: Evidence and Mechanisms. Adv. Nutr..

[14] Naraki K., Ghasemzadeh Rahbardar M., Ajiboye B.O., Hosseinzadeh H. (2023). The Effect of Ellagic Acid on the Metabolic Syndrome: A Review Article. Heliyon.

[15] Zhao L., Mehmood A., Soliman M.M., Iftikhar A., Iftikhar M., Aboelenin S.M., Wang C. (2021). Protective Effects of Ellagic Acid Against Alcoholic Liver Disease in Mice. Front. Nutr..

[16] Agrawal O.D., Kulkarni Y.A. (2020). Mini-Review of Analytical Methods Used in Quantification of Ellagic Acid. Rev. Anal. Chem..

[17] Ramírez de Molina A., Vargas T., Molina S., Sánchez J., Martínez-Romero J., González-Vallinas M., Martín-Hernández R., Sánchez-Martínez R., Gómez de Cedrón M., Dávalos A. (2015). The Ellagic Acid Derivative 4,4’-Di-O-Methylellagic Acid Efficiently Inhibits Colon Cancer Cell Growth through a Mechanism Involving WNT16. J. Pharmacol. Exp. Ther..

[18] Siraj M.A., Shilpi J.A., Hossain M.G., Uddin S.J., Islam M.K., Jahan I.A., Hossain H. (2016). Anti-Inflammatory and Antioxidant Activity of Acalypha Hispida Leaf and Analysis of Its Major Bioactive Polyphenols by HPLC. Adv. Pharm. Bull..

[19] Tuyen P.T., Xuan T.D., Tu Anh T.T., Mai Van T., Ahmad A., Elzaawely A.A., Khanh T.D. (2018). Weed Suppressing Potential and Isolation of Potent Plant Growth Inhibitors from Castanea Crenata Sieb. et Zucc. Molecules.

[20] Sun Y., Liu J., Bayertai, Tang S., Zhou X. (2020). Analysis of Gallic Acid and Ellagic Acid in Leaves of Elaeagnus Angustifolia L. from Different Habitats and Times in Xinjiang by HPLC with Cluster Analysis. Acta Chromatogr..

[21] Falcão T.R., de Araújo A.A., Soares L.A.L., de Moraes Ramos R.T., Bezerra I.C.F., Ferreira M.R.A., de Souza Neto M.A., Melo M.C.N., de Araújo R.F., de Aguiar Guerra A.C.V. (2018). Crude Extract and Fractions from Eugenia Uniflora Linn Leaves Showed Anti-Inflammatory, Antioxidant, and Antibacterial Activities. BMC Complement. Altern. Med..

[22] Díaz-de-Cerio E., Arráez-Román D., Segura-Carretero A., Ferranti P., Nicoletti R., Perrotta G.M., Gómez-Caravaca A.M. (2018). Establishment of Pressurized-Liquid Extraction by Response Surface Methodology Approach Coupled to HPLC-DAD-TOF-MS for the Determination of Phenolic Compounds of Myrtle Leaves. Anal. Bioanal. Chem..

[23] Ifeanacho M.O., Ikewuchi C.C., Ikewuchi J.C. (2017). Investigation of the Profile of Phenolic Compounds in the Leaves and Stems of Pandiaka Heudelotii Using Gas Chromatography Coupled with Flame Ionization Detector. Food Sci. Nutr..

[24] Dhooghe L., Meert H., Cimanga R.K., Vlietinck A.J., Pieters L., Apers S. (2011). The Quantification of Ellagic Acid in the Crude Extract of Phyllanthus Amarus Schum. & Thonn. (Euphorbiaceae). Phytochem. Anal..

[25] Ghadage D.M., Kshirsagar P.R., Pai S.R., Chavan J.J. (2017). Extraction Efficiency, Phytochemical Profiles and Antioxidative Properties of Different Parts of Saptarangi (*Salacia chinensis* L.)—An Important Underutilized Plant. Biochem. Biophys. Rep..

[26] Sathyanarayanan S., Chandran R., Thankarajan S., Abrahamse H., Thangaraj P. (2018). Phytochemical Composition, Antioxidant and Anti-Bacterial Activity of Syzygium Calophyllifolium Walp. Fruit. J. Food Sci. Technol..

[27] Chun K.-S., Cha H.-H., Shin J.-W., Na H.-K., Park K.-K., Chung W.-Y., Surh Y.-J. (2004). Nitric Oxide Induces Expression of Cyclooxygenase-2 in Mouse Skin through Activation of NF-κB. Carcinogenesis.

[28] González-Sarrías A., Larrosa M., Tomás-Barberán F.A., Dolara P., Espín J.C. (2010). NF-κB-Dependent Anti-Inflammatory Activity of Urolithins, Gut Microbiota Ellagic Acid-Derived Metabolites, in Human Colonic Fibroblasts. Br. J. Nutr..

[29] Hassoun E.A., Walter A.C., Alsharif N.Z., Stohs S.J. (1997). Modulation of TCDD-Induced Fetotoxicity and Oxidative Stress in Embryonic and Placental Tissues of C57BL/6J Mice by Vitamin E Succinate and Ellagic Acid. Toxicology.

[30] Amić D., Stepanić V., Lučić B., Marković Z., Dimitrić Marković J.M. (2013). PM6 Study of Free Radical Scavenging Mechanisms of Flavonoids: Why Does O-H Bond Dissociation Enthalpy Effectively Represent Free Radical Scavenging Activity?. J. Mol. Model..

[31] Mazzone G., Toscano M., Russo N. (2013). Density Functional Predictions of Antioxidant Activity and UV Spectral Features of Nasutin A, Isonasutin, Ellagic Acid, and One of Its Possible Derivatives. J. Agric. Food Chem..

[32] Kumar A., Kaushik P., Incerpi S., Pedersen J.Z., Goel S., Prasad A.K., Rohil V., Parmar V.S., Saso L., Len C. (2021). Evaluation of the Free Radical Scavenging Activities of Ellagic Acid and Ellagic Acid Peracetate by EPR Spectrometry. Molecules.

[33] Przewloka S.R., Shearer B.J. (2002). The Further Chemistry of Ellagic Acid II. Ellagic Acid and Water-Soluble Ellagates as Metal Precipitants. Holzforschung.

[34] Dalvi L.T., Moreira D.C., Andrade R., Ginani J., Alonso A., Hermes-Lima M. (2017). Ellagic Acid Inhibits Iron-Mediated Free Radical Formation. Spectrochim. Acta A Mol. Biomol. Spectrosc..

[35] Hwang J.M., Cho J.S., Kim T.H., Lee Y.I. (2010). Ellagic Acid Protects Hepatocytes from Damage by Inhibiting Mitochondrial Production of Reactive Oxygen Species. Biomed. Pharmacother..

[36] Hseu Y.-C., Chou C.-W., Senthil Kumar K.J., Fu K.-T., Wang H.-M., Hsu L.-S., Kuo Y.-H., Wu C.-R., Chen S.-C., Yang H.-L. (2012). Ellagic Acid Protects Human Keratinocyte (HaCaT) Cells against UVA-Induced Oxidative Stress and Apoptosis through the Upregulation of the HO-1 and Nrf-2 Antioxidant Genes. Food Chem. Toxicol..

[37] Duan M., Xiang Z., Xu H. (2024). Progress in Anti-Inflammatory Effect of Ellagic Acid. Med. Res..

[38] Nworu C.S., Akah P.A. (2015). ANTI-INFLAMMATORY HERBS AND THEIR MOLECULAR MECHANISMS OF ACTION. Afr. J. Tradit. Complement. Altern. Med..

[39] Cornélio Favarin D., Martins Teixeira M., Lemos de Andrade E., de Freitas Alves C., Lazo Chica J.E., Artério Sorgi C., Faccioli L.H., Paula Rogerio A. (2013). Anti-Inflammatory Effects of Ellagic Acid on Acute Lung Injury Induced by Acid in Mice. Mediat. Inflamm..

[40] Mansouri M.T., Hemmati A.A., Naghizadeh B., Mard S.A., Rezaie A., Ghorbanzadeh B. (2015). A Study of the Mechanisms Underlying the Anti-Inflammatory Effect of Ellagic Acid in Carrageenan-Induced Paw Edema in Rats. Indian J. Pharmacol..

[41] Marín M., María Giner R., Ríos J.-L., Recio M.C. (2013). Intestinal Anti-Inflammatory Activity of Ellagic Acid in the Acute and Chronic Dextrane Sulfate Sodium Models of Mice Colitis. J. Ethnopharmacol..

[42] Allahverdi T.D., Allahverdi E., Yayla S., Deprem T., Merhan O., Vural S. (2014). The Comparison of the Effects of Ellagic Acid and Diclofenac Sodium on Intra-Abdominal Adhesion: An In Vivo Study in the Rat Model. Int. Surg..

[43] Sarkar S., Siddiqui A.A., Mazumder S., De R., Saha S.J., Banerjee C., Iqbal M.S., Adhikari S., Alam A., Roy S. (2015). Ellagic Acid, a Dietary Polyphenol, Inhibits Tautomerase Activity of Human Macrophage Migration Inhibitory Factor and Its Pro-Inflammatory Responses in Human Peripheral Blood Mononuclear Cells. J. Agric. Food Chem..

[44] Lin Z., Lin C., Fu C., Lu H., Jin H., Chen Q., Pan J. (2020). The Protective Effect of Ellagic Acid (EA) in Osteoarthritis: An in Vitro and in Vivo Study. Biomed. Pharmacother..

[45] Garza-Lombó C., Posadas Y., Quintanar L., Gonsebatt M.E., Franco R. (2018). Neurotoxicity Linked to Dysfunctional Metal Ion Homeostasis and Xenobiotic Metal Exposure: Redox Signaling and Oxidative Stress. Antioxid. Redox Signal.

[46] Adamu A., Li S., Gao F., Xue G. (2024). The Role of Neuroinflammation in Neurodegenerative Diseases: Current Understanding and Future Therapeutic Targets. Front. Aging Neurosci..

[47] Javaid N., Shah M.A., Rasul A., Chauhdary Z., Saleem U., Khan H., Ahmed N., Uddin M.S., Mathew B., Behl T. (2021). Neuroprotective Effects of Ellagic Acid in Alzheimer’s Disease: Focus on Underlying Molecular Mechanisms of Therapeutic Potential. Curr. Pharm. Des..

[48] Zhu H., Yan Y., Jiang Y., Meng X. (2022). Ellagic Acid and Its Anti-Aging Effects on Central Nervous System. Int. J. Mol. Sci..

[49] Goudarzi M., Amiri S., Nesari A., Hosseinzadeh A., Mansouri E., Mehrzadi S. (2018). The Possible Neuroprotective Effect of Ellagic Acid on Sodium Arsenate-Induced Neurotoxicity in Rats. Life Sci..

[50] Ozben T., Ozben S. (2019). Neuro-Inflammation and Anti-Inflammatory Treatment Options for Alzheimer’s Disease. Clin. Biochem..

[51] Sanadgol N., Golab F., Mostafaie A., Mehdizadeh M., Abdollahi M., Sharifzadeh M., Ravan H. (2016). Ellagic Acid Ameliorates Cuprizone-Induced Acute CNS Inflammation via Restriction of Microgliosis and down-Regulation of CCL2 and CCL3 pro-Inflammatory Chemokines. Cell Mol. Biol..

[52] Kaur R., Mehan S., Khanna D., Kalra S., Parveen S. (2015). Precautionary Ellagic Acid Treatment Ameliorates Chronically Administered Scopolamine Induced Alzheimer’s Type Memory and Cognitive Dysfunctions in Rats. Pharmacologia.

[53] Chowdhury S., Kumar S. (2021). Inhibition of BACE1, MAO-B, Cholinesterase Enzymes, and Anti-Amyloidogenic Potential of Selected Natural Phytoconstituents: Multi-Target-Directed Ligand Approach. J. Food Biochem..

[54] Ardah M.T., Bharathan G., Kitada T., Haque M.E. (2020). Ellagic Acid Prevents Dopamine Neuron Degeneration from Oxidative Stress and Neuroinflammation in MPTP Model of Parkinson’s Disease. Biomolecules.

[55] Yee L.C., Adrian C.W., Khamki K.A., Ying K.Y., Shahfri M.F.M., Hee N.B., Nelly N.M.N., Santhirasaygaran P., Han S.Y., Yi T.C. (2023). Physical Health Impacts of Obesity: Comprehensive Review. Prog. Drug Discov. Biomed. Sci..

[56] Kubota S., Tanaka Y., Nagaoka S. (2019). Ellagic Acid Affects mRNA Expression Levels of Genes That Regulate Cholesterol Metabolism in HepG2 Cells. Biosci. Biotechnol. Biochem..

[57] Mejia-Meza E.I., Yáñez J.A., Remsberg C.M., Takemoto J.K., Davies N.M., Rasco B., Clary C. (2010). Effect of Dehydration on Raspberries: Polyphenol and Anthocyanin Retention, Antioxidant Capacity, and Antiadipogenic Activity. J. Food Sci..

[58] Wang L., Li L., Ran X., Long M., Zhang M., Tao Y., Luo X., Wang Y., Ma X., Halmurati U. (2013). Ellagic Acid Reduces Adipogenesis through Inhibition of Differentiation-Prevention of the Induction of Rb Phosphorylation in 3T3-L1 Adipocytes. Evid. Based Complement. Altern. Med..

[59] Makino-Wakagi Y., Yoshimura Y., Uzawa Y., Zaima N., Moriyama T., Kawamura Y. (2012). Ellagic Acid in Pomegranate Suppresses Resistin Secretion by a Novel Regulatory Mechanism Involving the Degradation of Intracellular Resistin Protein in Adipocytes. Biochem. Biophys. Res. Commun..

[60] Wang L., Wei Y., Ning C., Zhang M., Fan P., Lei D., Du J., Gale M., Ma Y., Yang Y. (2019). Ellagic Acid Promotes Browning of White Adipose Tissues in High-Fat Diet-Induced Obesity in Rats through Suppressing White Adipocyte Maintaining Genes. Endocr. J..

[61] Kim K.J., Jeong E.-S., Lee K.H., Na J.-R., Park S., Kim J.S., Na C.-S., Kim Y.R., Kim S. (2020). Unripe Rubus Coreanus Miquel Extract Containing Ellagic Acid Promotes Lipolysis and Thermogenesis In Vitro and In Vivo. Molecules.

[62] Ceci C., Graziani G., Faraoni I., Cacciotti I. (2020). Strategies to Improve Ellagic Acid Bioavailability: From Natural or Semisynthetic Derivatives to Nanotechnological Approaches Based on Innovative Carriers. Nanotechnology.

[63] Seeram N.P., Lee R., Heber D. (2004). Bioavailability of Ellagic Acid in Human Plasma after Consumption of Ellagitannins from Pomegranate (*Punica granatum* L.) Juice. Clin. Chim. Acta.

[64] Zuccari G., Baldassari S., Ailuno G., Turrini F., Alfei S., Caviglioli G. (2020). Formulation Strategies to Improve Oral Bioavailability of Ellagic Acid. Appl. Sci..

[65] Mady F.M., Ibrahim S.R.-M. (2018). Cyclodextrin-Based Nanosponge for Improvement of Solubility and Oral Bioavailability of Ellagic Acid. Pak. J. Pharm. Sci..

[66] Loo W.T., Jin L.J., Cheung M.N., Chow L.W. (2010). Evaluation of Ellagic Acid on the Activities of Oral Bacteria with the Use of Adenosine Triphosphate (ATP) Bioluminescence Assay. AJB.

[67] Ambrose S.S., Solairaj P., Subramoniam A. (2013). Effectiveness of Ellagic Acid on Isoniazid-Rifampicin Induced Liver Damage in Rats. J. Pharmacol. Pharmacother..

[68] Trefts E., Gannon M., Wasserman D.H. (2017). The Liver. Curr. Biol..

[69] Chiang D.J., McCullough A.J. (2014). The Impact of Obesity and Metabolic Syndrome on Alcoholic Liver Disease. Clin. Liver Dis..

[70] Pimpin L., Cortez-Pinto H., Negro F., Corbould E., Lazarus J.V., Webber L., Sheron N. (2018). Burden of Liver Disease in Europe: Epidemiology and Analysis of Risk Factors to Identify Prevention Policies. J. Hepatol..

[71] Zhong C., Qiu S., Li J., Shen J., Zu Y., Shi J., Sui G. (2019). Ellagic Acid Synergistically Potentiates Inhibitory Activities of Chemotherapeutic Agents to Human Hepatocellular Carcinoma. Phytomedicine.

[72] Keshtzar E., Khodayar M.J., Javadipour M., Ghaffari M.A., Bolduc D.L., Rezaei M. (2016). Ellagic Acid Protects against Arsenic Toxicity in Isolated Rat Mitochondria Possibly through the Maintaining of Complex II. Hum. Exp. Toxicol..

[73] Athukuri B.L., Neerati P. (2016). Enhanced Oral Bioavailability of Metoprolol with Gallic Acid and Ellagic Acid in Male Wistar Rats: Involvement of CYP2D6 Inhibition. Drug Metab. Pers. Ther..

[74] Ding X., Jian T., Wu Y., Zuo Y., Li J., Lv H., Ma L., Ren B., Zhao L., Li W. (2019). Ellagic Acid Ameliorates Oxidative Stress and Insulin Resistance in High Glucose-Treated HepG2 Cells via miR-223/Keap1-Nrf2 Pathway. Biomed. Pharmacother..

[75] Navarro M., Amigo-Benavent M., Mesias M., Baeza G., Gökmen V., Bravo L., Morales F.J. (2014). An Aqueous Pomegranate Seed Extract Ameliorates Oxidative Stress of Human Hepatoma HepG2 Cells. J. Sci. Food Agric..

[76] Priyadarsini K.I., Khopde S.M., Kumar S.S., Mohan H. (2002). Free Radical Studies of Ellagic Acid, a Natural Phenolic Antioxidant. J. Agric. Food Chem..

[77] Abdelkader N.F., Elyamany M., Gad A.M., Assaf N., Fawzy H.M., Elesawy W.H. (2020). Ellagic Acid Attenuates Liver Toxicity Induced by Valproic Acid in Rats. J. Pharmacol. Sci..

[78] Celik G., Semiz A., Karakurt S., Arslan S., Adali O., Sen A. (2013). A Comparative Study for the Evaluation of Two Doses of Ellagic Acid on Hepatic Drug Metabolizing and Antioxidant Enzymes in the Rat. BioMed Res. Int..

[79] Block K.I., Koch A.C., Mead M.N., Tothy P.K., Newman R.A., Gyllenhaal C. (2008). Impact of Antioxidant Supplementation on Chemotherapeutic Toxicity: A Systematic Review of the Evidence from Randomized Controlled Trials. Int. J. Cancer.

[80] Biswas S.K. (2016). Does the Interdependence between Oxidative Stress and Inflammation Explain the Antioxidant Paradox?. Oxidative Med. Cell. Longev..

[81] Kuhad A., Chopra K. (2009). Attenuation of Diabetic Nephropathy by Tocotrienol: Involvement of NFkB Signaling Pathway. Life Sci..

[82] Ahad A., Ganai A.A., Mujeeb M., Siddiqui W.A. (2014). Ellagic Acid, an NF-κB Inhibitor, Ameliorates Renal Function in Experimental Diabetic Nephropathy. Chem. -Biol. Interact..

[83] Winand J., Schneider Y.-J. (2014). The Anti-Inflammatory Effect of a Pomegranate Husk Extract on Inflamed Adipocytes and Macrophages Cultivated Independently, but Not on the Inflammatory Vicious Cycle between Adipocytes and Macrophages. Food Funct..

[84] Seo C.-S., Jeong S.-J., Yoo S.-R., Lee N.-R., Shin H.-K. (2016). Quantitative Analysis and In Vitro Anti-Inflammatory Effects of Gallic Acid, Ellagic Acid, and Quercetin from Radix Sanguisorbae. Pharmacogn. Mag..

[85] Banerjee R.R., Rangwala S.M., Shapiro J.S., Rich A.S., Rhoades B., Qi Y., Wang J., Rajala M.W., Pocai A., Scherer P.E. (2004). Regulation of Fasted Blood Glucose by Resistin. Science.

[86] Yoshimura Y., Nishii S., Zaima N., Moriyama T., Kawamura Y. (2013). Ellagic Acid Improves Hepatic Steatosis and Serum Lipid Composition through Reduction of Serum Resistin Levels and Transcriptional Activation of Hepatic *Ppara* in Obese, Diabetic KK-*Ay* Mice. Biochem. Biophys. Res. Commun..

[87] Yang Y.M., Kim S.Y., Seki E. (2019). Inflammation and Liver Cancer: Molecular Mechanisms and Therapeutic Targets. Semin. Liver Dis..

[88] Keenan B.P., Fong L., Kelley R.K. (2019). Immunotherapy in Hepatocellular Carcinoma: The Complex Interface between Inflammation, Fibrosis, and the Immune Response. J. Immunother. Cancer.

[89] Rønning S.B., Voldvik V., Bergum S.K., Aaby K., Borge G.I.A. (2020). Ellagic Acid and Urolithin A Modulate the Immune Response in LPS-Stimulated U937 Monocytic Cells and THP-1 Differentiated Macrophages. Food Funct..

[90] Du L., Li J., Zhang X., Wang L., Zhang W., Yang M., Hou C. (2019). Pomegranate Peel Polyphenols Inhibits Inflammation in LPS-Induced RAW264.7 Macrophages via the Suppression of TLR4/NF-κB Pathway Activation. Food Nutr. Res..

[91] Karimi M.Y., Fatemi I., Kalantari H., Mombeini M.A., Mehrzadi S., Goudarzi M. (2020). Ellagic Acid Prevents Oxidative Stress, Inflammation, and Histopathological Alterations in Acrylamide-Induced Hepatotoxicity in Wistar Rats. J. Diet. Suppl..

[92] Gu L., Deng W., Liu Y., Jiang C., Sun L., Sun X., Xu Q., Zhou H. (2014). Ellagic Acid Protects Lipopolysaccharide/d-Galactosamine-Induced Acute Hepatic Injury in Mice. Int. Immunopharmacol..

[93] Noori M., Jafari B., Hekmatdoost A. (2017). Pomegranate Juice Prevents Development of Non-Alcoholic Fatty Liver Disease in Rats by Attenuating Oxidative Stress and Inflammation. J. Sci. Food Agric..

[94] Labrecque L., Lamy S., Chapus A., Mihoubi S., Durocher Y., Cass B., Bojanowski M.W., Gingras D., Béliveau R. (2005). Combined Inhibition of PDGF and VEGF Receptors by Ellagic Acid, a Dietary-Derived Phenolic Compound. Carcinogenesis.

[95] Wang Y., Qiu Z., Zhou B., Liu C., Ruan J., Yan Q., Liao J., Zhu F. (2015). In Vitro Antiproliferative and Antioxidant Effects of Urolithin A, the Colonic Metabolite of Ellagic Acid, on Hepatocellular Carcinomas HepG2 Cells. Toxicol. Vitr..

[96] Ebrahimi R., Sepand M.R., Seyednejad S.A., Omidi A., Akbariani M., Gholami M., Sabzevari O. (2019). Ellagic Acid Reduces Methotrexate-Induced Apoptosis and Mitochondrial Dysfunction via up-Regulating Nrf2 Expression and Inhibiting the IĸBα/NFĸB in Rats. DARU J. Pharm. Sci..

[97] Aslan A., Gok O., Erman O., Kuloglu T. (2018). Ellagic Acid Impedes Carbontetrachloride-Induced Liver Damage in Rats through Suppression of NF-kB, Bcl-2 and Regulating Nrf-2 and Caspase Pathway. Biomed. Pharmacother..

[98] Shendge A.K., Basu T., Panja S., Chaudhuri D., Mandal N. (2018). An Ellagic Acid Isolated from *Clerodendrum Viscosum* Leaves Ameliorates Iron-Overload Induced Hepatotoxicity in Swiss Albino Mice through Inhibition of Oxidative Stress and the Apoptotic Pathway. Biomed. Pharmacother..

[99] Mishra S., Vinayak M. (2015). Role of Ellagic Acid in Regulation of Apoptosis by Modulating Novel and Atypical PKC in Lymphoma Bearing Mice. BMC Complement. Altern. Med..

[100] Mehal W.Z., Schuppan D. (2015). Antifibrotic Therapies in the Liver. Semin. Liver Dis..

[101] Buniatian G.H., Weiskirchen R., Weiss T.S., Schwinghammer U., Fritz M., Seferyan T., Proksch B., Glaser M., Lourhmati A., Buadze M. (2020). Antifibrotic Effects of Amyloid-Beta and Its Loss in Cirrhotic Liver. Cells.

[102] Higashi T., Friedman S.L., Hoshida Y. (2017). Hepatic Stellate Cells as Key Target in Liver Fibrosis. Adv. Drug Deliv. Rev..

[103] Yoshida K., Murata M., Yamaguchi T., Matsuzaki K., Okazaki K. (2016). Reversible Human TGF-β Signal Shifting between Tumor Suppression and Fibro-Carcinogenesis: Implications of Smad Phospho-Isoforms for Hepatic Epithelial-Mesenchymal Transitions. J. Clin. Med..

[104] Lee J.H., Won J.H., Choi J.M., Cha H.H., Jang Y.J., Park S., Kim H.G., Kim H.C., Kim D.K. (2014). Protective Effect of Ellagic Acid on Concanavalin A-Induced Hepatitis via Toll-Like Receptor and Mitogen-Activated Protein Kinase/Nuclear Factor κB Signaling Pathways. J. Agric. Food Chem..

[105] Panchal S.K., Ward L., Brown L. (2013). Ellagic Acid Attenuates High-Carbohydrate, High-Fat Diet-Induced Metabolic Syndrome in Rats. Eur. J. Nutr..

[106] Nankar R.P., Doble M. (2017). Hybrid Drug Combination: Anti-Diabetic Treatment of Type 2 Diabetic Wistar Rats with Combination of Ellagic Acid and Pioglitazone. Phytomedicine.

[107] Chao P.-C., Hsu C.-C., Yin M.-C. (2009). Anti-Inflammatory and Anti-Coagulatory Activities of Caffeic Acid and Ellagic Acid in Cardiac Tissue of Diabetic Mice. Nutr. Metab..

[108] Zhang C., Hu J., Sheng L., Yuan M., Wu Y., Chen L., Wang G., Qiu Z. (2019). Ellagic Acid Ameliorates AKT-Driven Hepatic Steatosis in Mice by Suppressing de Novo Lipogenesis via the AKT/SREBP-1/FASN Pathway. Food Funct..

[109] Goyal Y., Koul A., Ranawat P. (2019). Ellagic Acid Ameliorates Cisplatin Induced Hepatotoxicity in Colon Carcinogenesis. Environ. Toxicol..

[110] Guo S., Ren X., He K., Chen X., Zhang S., Roller M., Zheng B., Zheng Q., Ho C.-T., Bai N. (2020). The Anti-Diabetic Effect of Eight Lagerstroemia Speciosa Leaf Extracts Based on the Contents of Ellagitannins and Ellagic Acid Derivatives. Food Funct..

[111] Polce S.A., Burke C., França L.M., Kramer B., Paes A.M.d.A., Carrillo-Sepulveda M.A. (2018). Ellagic Acid Alleviates Hepatic Oxidative Stress and Insulin Resistance in Diabetic Female Rats. Nutrients.

[112] Fatima N., Hafizur R.M., Hameed A., Ahmed S., Nisar M., Kabir N. (2017). Ellagic Acid in Emblica Officinalis Exerts Anti-Diabetic Activity through the Action on β-Cells of Pancreas. Eur. J. Nutr..

[113] Ghadimi M., Foroughi F., Hashemipour S., Rashidi Nooshabadi M., Ahmadi M.H., Ahadi Nezhad B., Khadem Haghighian H. (2021). Randomized Double-Blind Clinical Trial Examining the Ellagic Acid Effects on Glycemic Status, Insulin Resistance, Antioxidant, and Inflammatory Factors in Patients with Type 2 Diabetes. Phytother. Res..

[114] González-Sarrías A., García-Villalba R., Núñez-Sánchez M.Á., Tomé-Carneiro J., Zafrilla P., Mulero J., Tomás-Barberán F.A., Espín J.C. (2015). Identifying the Limits for Ellagic Acid Bioavailability: A Crossover Pharmacokinetic Study in Healthy Volunteers after Consumption of Pomegranate Extracts. J. Funct. Foods.

[115] Zhang M., Cui S., Mao B., Zhang Q., Zhao J., Zhang H., Tang X., Chen W. (2023). Ellagic Acid and Intestinal Microflora Metabolite Urolithin A: A Review on Its Sources, Metabolic Distribution, Health Benefits, and Biotransformation. Crit. Rev. Food Sci. Nutr..

[116] Xu H., Lv D., Guan Y. (2025). Appeal of Urolithins from Synthesis to Biological Activities. J. Agric. Food Chem..

[117] Tomás-Barberán F.A., García-Villalba R., González-Sarrías A., Selma M.V., Espín J.C. (2014). Ellagic Acid Metabolism by Human Gut Microbiota: Consistent Observation of Three Urolithin Phenotypes in Intervention Trials, Independent of Food Source, Age, and Health Status. J. Agric. Food Chem..

[118] Zhang H., Li C., Han L., Xiao Y., Bian J., Liu C., Gong L., Liu Z., Wang M. (2024). MUP1 Mediates Urolithin A Alleviation of Chronic Alcohol-Related Liver Disease via Gut-Microbiota-Liver Axis. Gut Microbes.

[119] Luo J., Yang Y., Liu H., Tan Z., Chen C., Li W., Yang R. (2025). Ellagic Acid Alleviates High-Fructose Diet-Induced Non-Alcoholic Fatty Liver Disease by Modulating Liver Metabolic Profiles and Gut Microbiota. Int. J. Food Sci. Nutr..

[120] Li X., He M., Yi X., Lu X., Zhu M., Xue M., Tang Y., Zhu Y. (2024). Short-Chain Fatty Acids in Nonalcoholic Fatty Liver Disease: New Prospects for Short-Chain Fatty Acids as Therapeutic Targets. Heliyon.

[121] Zhang D., Jian Y.-P., Zhang Y.-N., Li Y., Gu L.-T., Sun H.-H., Liu M.-D., Zhou H.-L., Wang Y.-S., Xu Z.-X. (2023). Short-Chain Fatty Acids in Diseases. Cell Commun. Signal..

[122] Leng P., Wang Y., Xie M. (2025). Ellagic Acid and Gut Microbiota: Interactions, and Implications for Health. Food Sci. Nutr..

[123] Li H., Liang J., Han M., Gao Z. (2025). Polyphenols Synergistic Drugs to Ameliorate Non-Alcoholic Fatty Liver Disease via Signal Pathway and Gut Microbiota: A Review. J. Adv. Res..

[124] Guo X., Yin X., Liu Z., Wang J. (2022). Non-Alcoholic Fatty Liver Disease (NAFLD) Pathogenesis and Natural Products for Prevention and Treatment. Int. J. Mol. Sci..

[125] Day C.P. (2005). Natural History of NAFLD: Remarkably Benign in the Absence of Cirrhosis. Gastroenterology.

[126] Sinn D.H., Kang D., Guallar E., Choi S.C., Hong Y.S., Park Y., Cho J., Gwak G.-Y. (2023). Regression of Nonalcoholic Fatty Liver Disease Is Associated with Reduced Risk of Incident Diabetes: A Longitudinal Cohort Study. PLoS ONE.

[127] Ilyas F., Ali H., Patel P., Sarfraz S., Basuli D., Giammarino A., Satapathy S.K. (2023). Increasing Nonalcoholic Fatty Liver Disease–Related Mortality Rates in the United States from 1999 to 2022. Hepatol. Commun..

[128] Teng M.L., Ng C.H., Huang D.Q., Chan K.E., Tan D.J., Lim W.H., Yang J.D., Tan E., Muthiah M.D. (2023). Global Incidence and Prevalence of Nonalcoholic Fatty Liver Disease. Clin. Mol. Hepatol..

[129] Paik J.M., Henry L., Younossi Y., Ong J., Alqahtani S., Younossi Z.M. (2023). The Burden of Nonalcoholic Fatty Liver Disease (NAFLD) Is Rapidly Growing in Every Region of the World from 1990 to 2019. Hepatol. Commun..

[130] Rinella M.E., Lazarus J.V., Ratziu V., Francque S.M., Sanyal A.J., Kanwal F., Romero D., Abdelmalek M.F., Anstee Q.M., Arab J.P. (2023). A Multisociety Delphi Consensus Statement on New Fatty Liver Disease Nomenclature. Hepatology.

[131] Tilg H., Adolph T.E., Moschen A.R. (2021). Multiple Parallel Hits Hypothesis in Nonalcoholic Fatty Liver Disease: Revisited After a Decade. Hepatology.

[132] Buzzetti E., Pinzani M., Tsochatzis E.A. (2016). The Multiple-Hit Pathogenesis of Non-Alcoholic Fatty Liver Disease (NAFLD). Metabolism.

[133] Wainwright P., Byrne C.D. (2016). Bidirectional Relationships and Disconnects between NAFLD and Features of the Metabolic Syndrome. Int. J. Mol. Sci..

[134] Kim J.-S., Song B.-J., Cho Y.-E. (2025). Pomegranate-Derived Exosome-Like Nanovesicles Containing Ellagic Acid Alleviate Gut Leakage and Liver Injury in MASLD. Food Sci. Nutr..

[135] Ren S.-M., Zhang Q.-Z., Chen M.-L., Jiang M., Zhou Y., Xu X.-J., Wang D.-M., Pan Y.-N., Liu X.-Q. (2021). Anti-NAFLD Effect of Defatted Walnut Powder Extract in High Fat Diet-Induced C57BL/6 Mice by Modulating the Gut Microbiota. J. Ethnopharmacol..

[136] Xu J., Tian H., Ji Y., Dong L., Liu Y., Wang Y., Gao X., Shi H., Li H., Yang L. (2023). Urolithin C Reveals Anti-NAFLD Potential via AMPK-Ferroptosis Axis and Modulating Gut Microbiota. Naunyn Schmiedebergs Arch. Pharmacol..

[137] Leung C., Rivera L., Furness J.B., Angus P.W. (2016). The Role of the Gut Microbiota in NAFLD. Nat. Rev. Gastroenterol. Hepatol..

[138] Elseweidy M.M., Elesawy A.E., Sobh M.S., Elnagar G.M. (2022). Ellagic Acid Ameliorates High Fructose-Induced Hyperuricemia and Non-Alcoholic Fatty Liver in Wistar Rats: Focusing on the Role of C1q/Tumor Necrosis Factor-Related Protein-3 and ATP Citrate Lyase. Life Sci..

[139] Mighani S., Samimi R., Nooshabadi M.R., Farzam S.A., Haghighian H.K., Javadi M. (2025). A Randomized Double-Blind Clinical Trial Investigating the Effects of Ellagic Acid on Glycemic Status, Liver Enzymes, and Oxidative Stress in Patients with Non-Alcoholic Fatty Liver Disease. BMC Complement. Med. Ther..

